# The Molecular Landscape of Placental Dysfunction in Fetal Growth Restriction (FGR): From Integrated Signaling Networks to Epigenetic Control

**DOI:** 10.3390/biology15212046

**Published:** 2026-11-09

**Authors:** Fani-Niki Varra, Michail Varras, Viktoria-Konstantina Varra, Panagiotis Theodosis-Nobelos, Konstantinos Stefanidis, Anastassios Philippou

**Affiliations:** 1 Department of Pharmacy, School of Health Sciences, Frederick University, Nicosia 1036, Cyprus; fannyvarra@gmail.com (F.-N.V.); hsc.np@frederick.ac.cy (P.T.-N.); 2 Medical School, Democritus University of Thrace, 6810 Alexandroupolis, Greece; 3 Department of Physiology, Medical School, National and Kapodistrian University of Athens, 11527 Athens, Greece; tfilipou@med.uoa.gr; 4 Fourth Department of Obstetrics and Gynecology, ‘Elena Venizelou’ General and Maternity Hospital, 11521 Athens, Greece; 5 Department of Pharmacy, School of Health Sciences, University of Patras, University Campus, 26504 Patra, Greece; nadv.ars2016@gmail.com; 6 First Department of Pathology, Medical School, National and Kapodistrian University of Athens, 11527 Athens, Greece; 7 “Advanced Aesthetics and Cosmetic Science: Development-Quality Control and Safety of New Cosmetic Products”, Division of Aesthetics and Cosmetic Science, Department of Biomedical Sciences, School of Health and Welfare Sciences, University of West Attica, Aigaleo, 12243 Athens, Greece; 8 First Department of Obstetrics and Gynecology, Alexandra General Hospital, Medical School, National and Kapodistrian University of Athens, 11528 Athens, Greece; kstefanidis@med.uoa.gr

**Keywords:** fetal growth restriction, placental insufficiency, NLRP3 inflammasome, angiogenesis, mTOR signaling, nutrient transport, spatial transcriptomics, targeted nanoparticles

## Abstract

Fetal growth restriction (FGR) is a major pregnancy complication linked to placental insufficiency, resulting from defective trophoblast differentiation, impaired maternal–fetal vascular adaptation, and altered nutrient sensing. This review synthesizes current evidence on the interconnected molecular networks driving placental dysfunction. We map the reciprocal crosstalk between the pro-inflammatory shift, highlighted by NLRP3 inflammasome-driven trophoblast pyroptosis, and dysregulated extracellular matrix remodeling mediated by the MMP-9/TIMP system. This hostile microenvironment feeds directly into the angiogenic imbalance defined by the sFlt-1/PlGF ratio, accelerating cellular senescence via p53 and disrupting survival hubs like the PTEN/PI3K/AKT axis. Consequently, downstream metabolic sensors, notably the mTOR pathway, are blunted, causing trafficking failure of essential amino acid (SNAT2, LAT1) and glucose (GLUT) transporters. Crucially, we introduce an original framework integrating emerging spatial transcriptomics and single-cell epigenomics, demonstrating that these pathomechanisms are anatomically regionalized rather than uniform. Finally, we provide a critical evaluation of translation-ready therapeutic targets, emphasizing that strict safety constraints against fetal teratogenesis render ligand-functionalized nanoparticles a non-negotiable prerequisite for placenta-selective drug delivery. Integrating molecular stratification with longitudinal biomarker profiling represents the next milestone for mechanism-based, targeted management of FGR.

## Simple Summary

Fetal growth restriction (FGR) occurs when a fetus does not achieve its biological growth potential, often due to placental insufficiency. This review summarizes the complex molecular networks driving placental dysfunction, highlighting how chronic inflammation, impaired vascular development, and epigenetic modifications collectively disrupt vital nutrient sensing and transport mechanisms. By integrating recent advances in single-cell epigenomics and spatial transcriptomics, we demonstrate that these pathological changes are anatomically regionalized across the placenta rather than uniform. Finally, we discuss how target-specific nanomedicine could safely deliver placenta-selective treatments without risking fetal toxicity. Ultimately, shifting the clinical focus toward a multi-marker network approach will pave the way for earlier detection, precise molecular stratification, and personalized management of FGR.

## 1. Introduction

Fetal growth restriction (FGR), traditionally termed intrauterine growth restriction (IUGR), describes a fetus that does not achieve its biological growth potential and is associated with increased risks of perinatal morbidity, mortality, and later-life disease [1,2]. Although estimated fetal weight or abdominal circumference below the 10th percentile is frequently used in clinical assessment, fetal smallness is not synonymous with pathological growth restriction; constitutionally small fetuses may be healthy, whereas FGR reflects an underlying pathological process [3,4]. FGR is multifactorial and may reflect maternal disease, fetal genetic or structural abnormalities, infection, environmental exposures, or placental pathology, with placental dysfunction representing a major mechanism in a substantial proportion of cases [5,6]. Normal placental function requires coordinated trophoblast differentiation and invasion, remodeling of the maternal spiral arteries, development of the villous vascular tree, and establishment of efficient maternal–fetal exchange. Inadequate placentation can leave the uteroplacental circulation with excessive vascular resistance and heterogeneous perfusion, reducing the capacity of the placenta to deliver oxygen and nutrients and to remove fetal metabolic waste [5,7].

FGR refers to a condition in which the sonographically estimated fetal weight (EFW) is below the 10th percentile for gestational age and is most commonly associated with placental dysfunction [1,3,4]. Appropriate-for-gestational-age (AGA) pregnancies were defined as uncomplicated pregnancies with an EFW between the 10th and 90th percentiles for gestational age. In contrast, small for gestational age (SGA) describes a newborn whose birth weight is below the 10th percentile for gestational age [1,3,4]. However, SGA does not necessarily indicate pathological growth restriction, as some fetuses are constitutionally small [1,3,4]. Although the terms IUGR and FGR are often used interchangeably in the literature [4], the term IUGR has largely been replaced by FGR. The principal concepts were combined. Structural and functional abnormalities of the trophoblast, villi, and fetoplacental vessels are therefore central to the pathophysiology of placental FGR [8]. Clinical surveillance combines serial assessment of fetal growth with Doppler velocimetry and other fetal well-being measures, while delivery is individualized according to gestational age, fetal condition, and the severity of placental dysfunction [2,4].

At the molecular level, FGR is best understood as a network disorder of the maternal–placental–fetal interface. Persistent inflammatory signaling can alter trophoblast proliferation, differentiation, invasion, and vascular function, while activation of the NLRP3 inflammasome may promote caspase-dependent inflammatory cell death and amplify placental injury [9]. In parallel, dysregulated extracellular matrix remodeling can interfere with trophoblast invasion and spiral artery remodeling. MMP-9 is particularly relevant because its activity contributes to matrix turnover and placental remodeling, although the direction and magnitude of its alteration appear to depend on tissue, gestational age, and FGR phenotype [10,11].

Angiogenic and growth factor pathways provide another major layer of regulation. VEGF and PlGF support placental vascular development, whereas sFlt-1 reduces the bioavailability of these pro-angiogenic factors; altered concentrations of PlGF and sFlt-1/PlGF have been associated with placental dysfunction and FGR [12,13,14]. TGF-β family signaling contributes to trophoblast differentiation and extracellular matrix organization, while the IGF/IGFBP system regulates trophoblast growth, survival, nutrient transport, and fetal growth. Cellular fate and chromatin dynamics are similarly important; p53 regulates cell-cycle arrest and apoptosis, PTEN constrains PI3K/AKT signaling, and ARID1A modifies chromatin accessibility and trophoblast transcriptional programs [15,16,17].

Crucially, the placenta acts as a dynamic mediator that links the maternal environment to the fetus through sophisticated epigenetic modifications and metabolic sensing pathways. Epigenetic and genomic regulation—encompassing DNA methylation, histone modifications, ATP-dependent chromatin remodeling, and non-coding RNAs—enables the fine-tuning of gene networks essential for trophoblast development and vascular adaptation [18,19]. When these epigenetic mechanisms are disrupted, placental metabolic signaling and nutrient transport are directly compromised. This includes severe alterations in key amino acid transport systems, such as System A (SNAT2) and System L (LAT1), as well as glucose transporter (GLUT) expression and trafficking, which collectively reduce the transport capacity of the placenta and restrict fetal growth [20,21]. Evidence from human placentas and experimental models suggests that these pathways do not operate in isolation but form an integrated, self-reinforcing pathological network [15,16,17].

The aim of this review is to provide a focused synthesis of the molecular mechanisms of placental dysfunction in FGR. We underscore the interplay among inflammation, extracellular matrix remodeling, angiogenesis, and growth factor signaling, while integrating recent insights into tumor suppressor pathways, epigenetic and genomic regulation, and placental metabolic and nutrient transport pathways. Finally, we discuss how these interconnected molecular networks may inform biomarker development and future mechanism-based therapeutic interventions.

## 2. Search Strategy

A structured narrative literature search was conducted to identify evidence on the molecular, cellular, and placental mechanisms involved in FGR, with emphasis on the biological pathways addressed in this review.

### 2.1. Information Sources and Search Period

Electronic searches were performed in PubMed/MEDLINE, Scopus, Web of Science, and Google Scholar for publications from 2000 through 30 September 2026. Reference lists of relevant reviews and primary studies were also screened to identify additional eligible publications contained within the existing bibliography of the original manuscript.

### 2.2. Core FGR and Placental Search Terms

The core search terms included “fetal growth restriction,” “FGR,” “fetal growth retardation,” “intrauterine growth restriction,” “IUGR,” “small for gestational age,” “SGA,” “fetal growth,” “placental insufficiency,” “placental dysfunction,” “placenta,” “trophoblast,” “maternal–fetal interface,” “maternal vascular malperfusion,” and “fetal vascular malperfusion”.

### 2.3. Molecular and Biological Domains

Inflammation and immune signaling were searched using “inflammation,” “placental inflammation,” “cytokines,” “TNF-α,” “IL-1β,” “IL-6,” “IL-10,” “NF-κB,” “NLRP3,” “inflammasome,” “caspase-1,” and “pyroptosis.” Extracellular matrix and trophoblast remodeling searches included “MMP-9,” “TIMP-1,” “extracellular matrix,” “trophoblast invasion,” “trophoblast migration,” “spiral artery remodeling,” and “placental remodeling”.

#### 2.3.1. Angiogenesis and Vascular Signaling

Searches included “angiogenesis,” “placental angiogenesis,” “VEGF,” “VEGFA,” “PlGF Bars,” “VEGFR,” “sFlt-1,” “soluble endoglin,” “vascular remodeling,” “endothelial dysfunction,” and “uteroplacental perfusion”.

#### 2.3.2. Growth Factor and Trophoblast Signaling

Searches included “TGF-α,” “TGF-β,” “TGF-β2,” “EGFR,” “insulin-like growth factor,” “IGF-1,” “IGF-2,” “IGF1R,” “IGF2R,” “IGFBP-1,” “IGFBP-2,” “IGFBP-3,” “IGFBP-4,” and “IGFBP-5”.

#### 2.3.3. Tumor Suppressor Pathways

Searches included “p53,” “TP53,” “PTEN,” and “ARID1A”.

#### 2.3.4. Metabolic Signaling and Nutrient Transport

Searches included “metabolic signaling,” “SNAT2,” “LAT1,” “GLUT1,” “GLUT3,” “GLUT4,” “nutrient transport,” and “amino acid transport”.

#### 2.3.5. Epigenetic and Genomic Regulation

Searches included “epigenetics,” “DNA methylation,” “histone modification,” “chromatin remodeling Bars,” “SWI/SNF,” “microRNA,” “miRNA,” “non-coding RNA,” “gene expression,” and “transcriptional regulation”.

### 2.4. Search Structure and Eligibility Criteria

The principal concepts were combined using the AND/OR Boolean operators, with phrase searching and truncation applied where supported by the database.

Eligible publications included human pregnancy studies, placental tissue analyses, studies of maternal or fetal biological samples, trophoblast or placental cell models, and relevant experimental animal studies. Studies were prioritized when they directly investigated FGR or placental insufficiency and provided mechanistic or clinical information relevant to the pathways addressed in this review.

Studies were considered eligible when they were relevant to the scope of the review and contributed evidence on the clinical, cellular, molecular, or experimental mechanisms associated with FGR or placental dysfunction. Publications were excluded when they were unrelated to the review objectives or did not provide relevant evidence for the pathways under consideration. Reference lists of relevant publications were examined to identify additional studies meeting these criteria. The language eligibility criteria followed those applied during the original literature selection.

### 2.5. Study Selection and Narrative Synthesis

Titles and abstracts were screened initially, followed by full-text assessment when appropriate. The selection process was guided by the relevance of each publication to the review objectives and the biological pathways discussed. Evidence was synthesized narratively and organized into specific clinical and molecular subsections: inflammation, extracellular matrix remodeling, angiogenesis, growth factor signaling, tumor suppressor pathways, epigenetic regulation, and placental metabolic signaling and nutrient transport. Human and experimental evidence were considered separately where appropriate, and mechanistic interpretations were not treated as equivalent to validated clinical biomarkers or established therapies.

### 2.6. Limitations of the Literature Search Subsection

Despite the broad literature search conducted across multiple databases, certain methodological limitations should be acknowledged. The search strategy was designed to identify and synthesize relevant evidence on the molecular and placental mechanisms underlying FGR; however, it was not conducted as a fully systematic review with a prospectively defined and fully reproducible screening protocol. Consequently, complete database-specific search strings, a quantitative account of records identified and excluded, and a formal record of the screening process were not consistently documented. Therefore, the possibility of selection bias and the omission of relevant studies cannot be excluded. These limitations should be considered when interpreting the findings, which are intended to provide a comprehensive narrative overview of the available evidence rather than an exhaustive systematic assessment.

## 3. Inflammation-Driven Placental Dysfunction in FGR

### 3.1. Cytokines as Mediators of Placental Inflammation and FGR

In recent years, increasing data indicate that alterations in maternal, fetal and placental inflammatory responses play a crucial role in the pathophysiology of FGR pregnancy [22]. Central to these processes are cytokines, a heterogeneous group of small secreted proteins (<40 kDa), which regulate the immune response through the activation, recruitment and differentiation of various types of immune cells. Cytokines are produced mainly by activated macrophages, but also by other cells of the immune system [23,24]. Based on their function, cytokines are divided into pro-inflammatory and anti-inflammatory. Pro-inflammatory cytokines include, among others, tumor necrosis factor-α (TNF-α), interleukin-1β (IL-1β), interleukin-6 (IL-6), interleukin-8 (IL-8) and interferon-γ (IFN-γ), which enhance the inflammatory response and contribute to the activation of innate and adaptive immune mechanisms [25]. In contrast, anti-inflammatory cytokines, such as interleukin-1 receptor antagonist (IL-1Ra), transforming growth factor-β (TGF-β), interleukin-4 (IL-4), interleukin-10 (IL-10), and interleukin-13 (IL-13), inhibit pro-inflammatory signals, contributing to the maintenance of immune balance [26].

**Figure 1:**
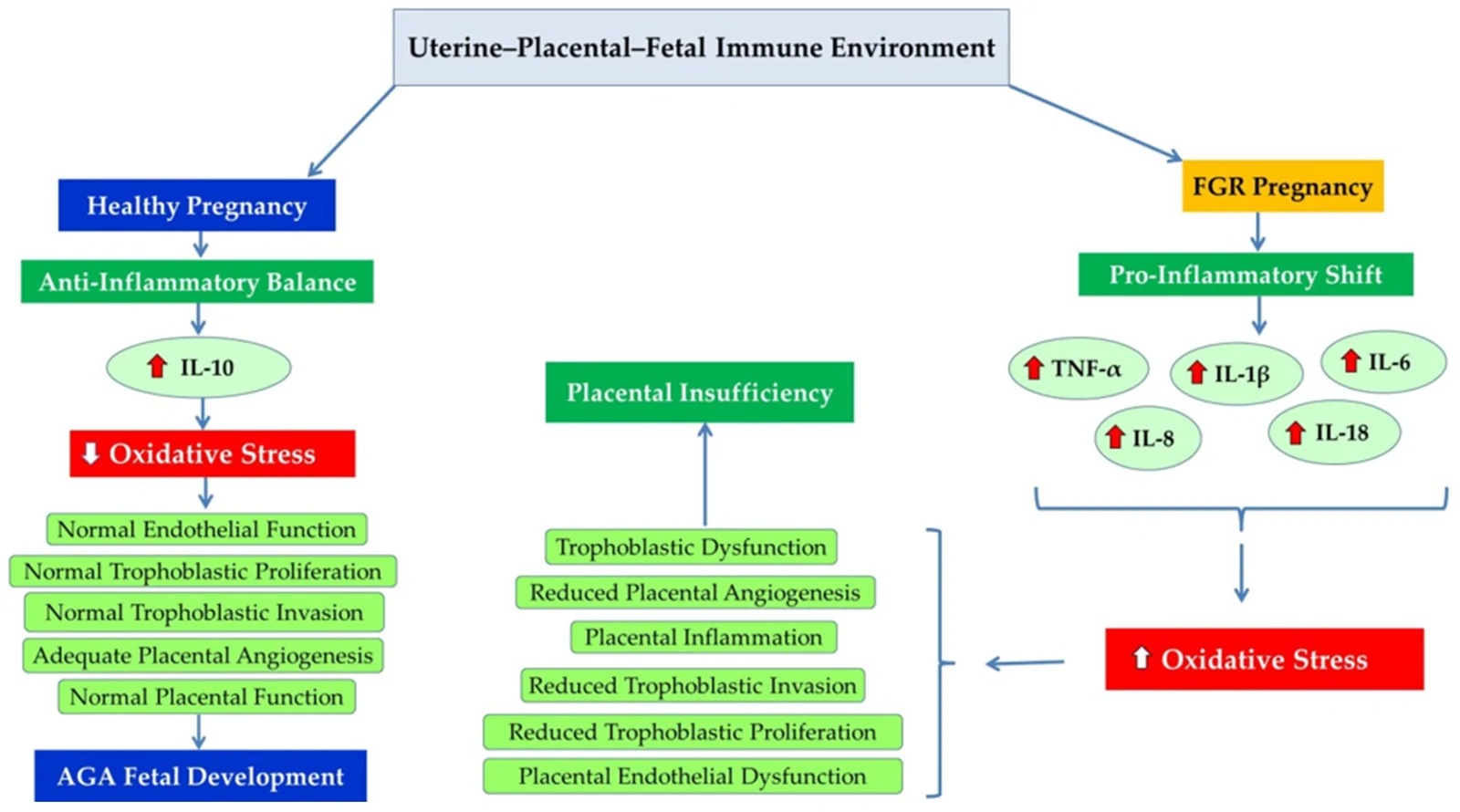
Schematic representation of the roles of pro-inflammatory and anti-inflammatory cytokines in regulating placental function during normal pregnancy and of immune imbalance contributing to the pathogenesis of FGR (abbreviations: IL-10: Ιnterleukin-10; TNF-α: Tumor necrosis factor-alpha; IL-1β: Interleukin-1 beta; IL-6: Interleukin-6; IL-8: Interleukin-8; IL-18: Interleukin-18; AGA: Appropriate for gestational age; FGR: Fetal growth restriction).

IL-10 is a key anti-inflammatory cytokine with a crucial role during pregnancy. In normal pregnancies, it contributes to maintaining the immune balance between mother and fetus, suppresses excessive inflammatory responses, and supports the integrity of the placental vascular network. Through these actions, it enhances the normal function of the placenta and promotes normal fetal development [27,28]. In contrast, in pregnancies complicated by FGR, a significant decrease in IL-10 levels has been reported in the maternal, fetal and placental environment, which is accompanied by increased expression of proinflammatory cytokines, such as TNF-α, IL-6, IL-8, IL-18 and IL-1β [28,29]. This imbalance between anti-inflammatory and pro-inflammatory factors is associated with the development of placental inflammation, impaired proliferation and invasive capacity of trophoblast cells, increased, and reduced placental vascularity [30,31] (Figure 1).

Overall, the available evidence suggests that dysregulation of the maternal–fetal immune balance in FGR promotes a pro-inflammatory placental environment that may impair placental function and compromise fetal growth and development. Further investigation into the interactions between pro-inflammatory and anti-inflammatory cytokines is expected to provide valuable insights into the identification of novel diagnostic biomarkers and the development of targeted preventive and therapeutic strategies. Nevertheless, a comprehensive understanding of the underlying molecular mechanisms and the establishment of the clinical efficacy of cytokine-targeted interventions require further experimental and clinical research.

### 3.2. The Emerging Role of Adipokines in Placental Dysfunction and FGR

Adipokines are bioactive proteins secreted mainly by adipose tissue and play an important role in the regulation of energy metabolism, glucose homeostasis and inflammatory processes. Based on their biological activity, they are distinguished into pro-inflammatory and anti-inflammatory adipokines [32]. The pro-inflammatory adipokines include leptin, visfatin, resistin and plasminogen activator inhibitor-1 (PAI-1), while adiponectin and apelin are among the adipokines with anti-inflammatory and cytoprotective properties [33].

During pregnancy, the progressive increase in fat mass and the accompanying metabolic and endocrine adaptations significantly affect the production and action of adipokines [34]. Thus, their study in pregnancies complicated by FGR is of particular interest, as it may contribute to the understanding of the underlying pathophysiological mechanisms and the identification of new biomarkers and therapeutic targets.

In healthy pregnancies, adiponectin participates in the regulation of nutrient transport across the placenta and influences cell signaling pathways that are essential for normal fetal development [35]. At the same time, apelin contributes to the regulation of angiogenesis, energy metabolism and inflammatory response, supporting the functional adequacy of the placenta and fetal development [36,37].

**Figure 2:**
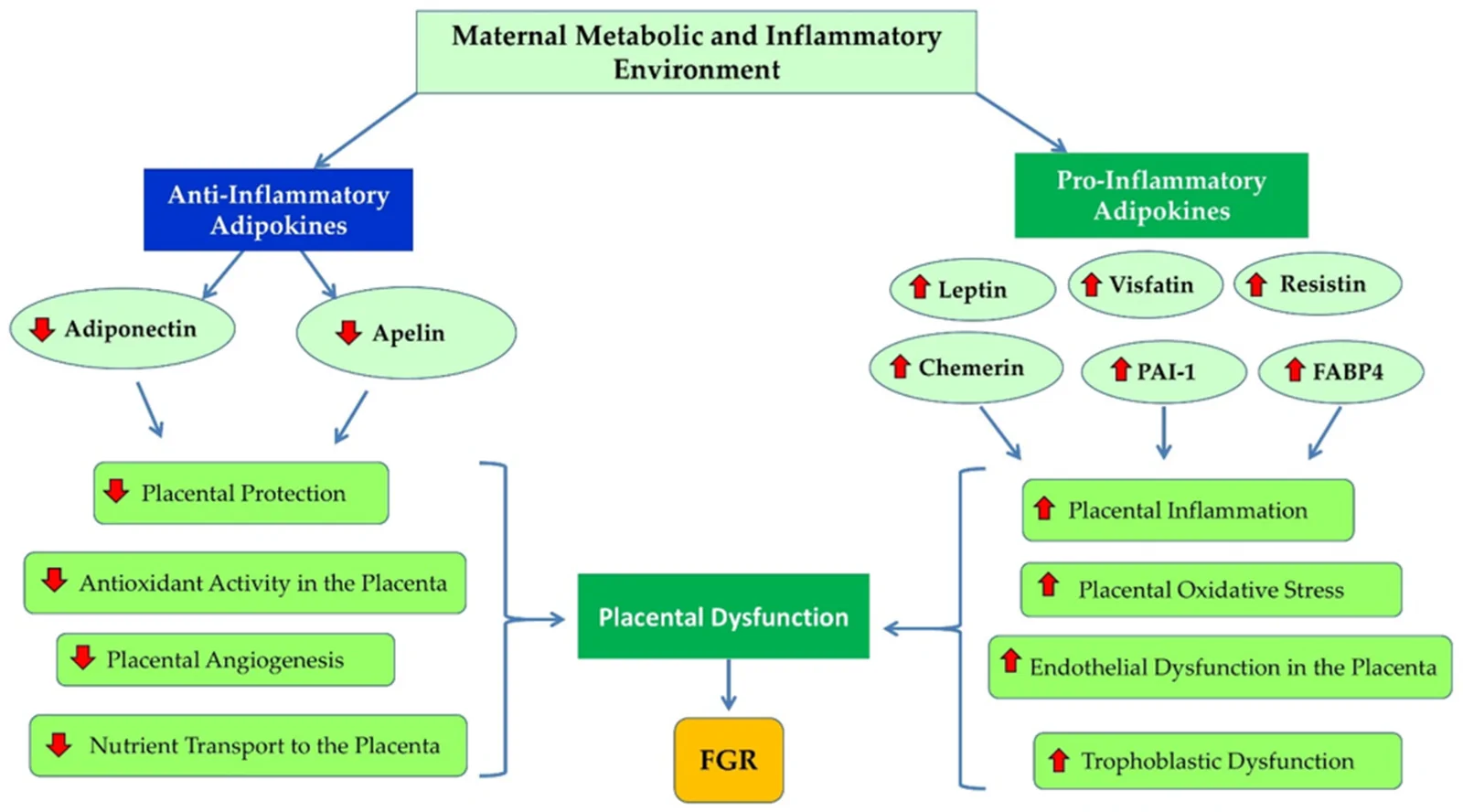
Schematic representation of the imbalance between anti-inflammatory adipokines (adiponectin and apelin) and pro-inflammatory adipokines (leptin, visfatin, resistin, PAI-1, chemerin, and FABP-4), and their contribution to the development of a pro-inflammatory placental microenvironment in FGR (abbreviations: PAI-1: Plasminogen Activator Inhibitor-1; FABP4: Fatty Acid Binding Protein 4; FGR: Fetal growth restriction).

In fetal growth restriction (FGR) pregnancies, reduced concentrations of both adiponectin and apelin have been reported in the maternal circulation and placental tissue compared to appropriate-for-gestational-age (AGA) controls [35,37]. This downregulation directly correlates with heightened inflammatory activity and impaired placental function [35,37] (Figure 2).

Conversely, several proinflammatory adipokines—including leptin, visfatin, resistin, plasminogen activator inhibitor-1 (PAI-1), chemerin, and fatty acid-binding protein 4 (FABP-4)—exhibit upregulated expression or elevated concentrations within the maternal and fetal circulation, as well as the placenta, during conditions of placental dysfunction and compromised fetal development [38]. These adipokines appear to exacerbate the inflammatory response, promote endothelial dysfunction, and adversely affect placental function, thereby contributing to the restriction of fetal growth [39,40] (Figure 2).

Overall, the available evidence indicates that pregnancies complicated by FGR are characterized by an imbalance between anti-inflammatory and pro-inflammatory adipokines, favoring the development of a pro-inflammatory placental microenvironment. The reduced expression of protective adipokines, together with the increased levels of pro-inflammatory mediators, may contribute to placental dysfunction and impaired fetal growth. Further investigation into the role of adipokines in the pathophysiology of FGR may facilitate the identification of novel diagnostic and prognostic biomarkers, as well as the development of innovative therapeutic strategies. However, additional experimental and clinical studies are needed to fully elucidate their biological roles and clinical significance.

### 3.3. NLRP3 Inflammasome Activation as a Driver of Inflammation, Pyroptosis, and Placental Dysfunction in FGR

**Figure 3:**
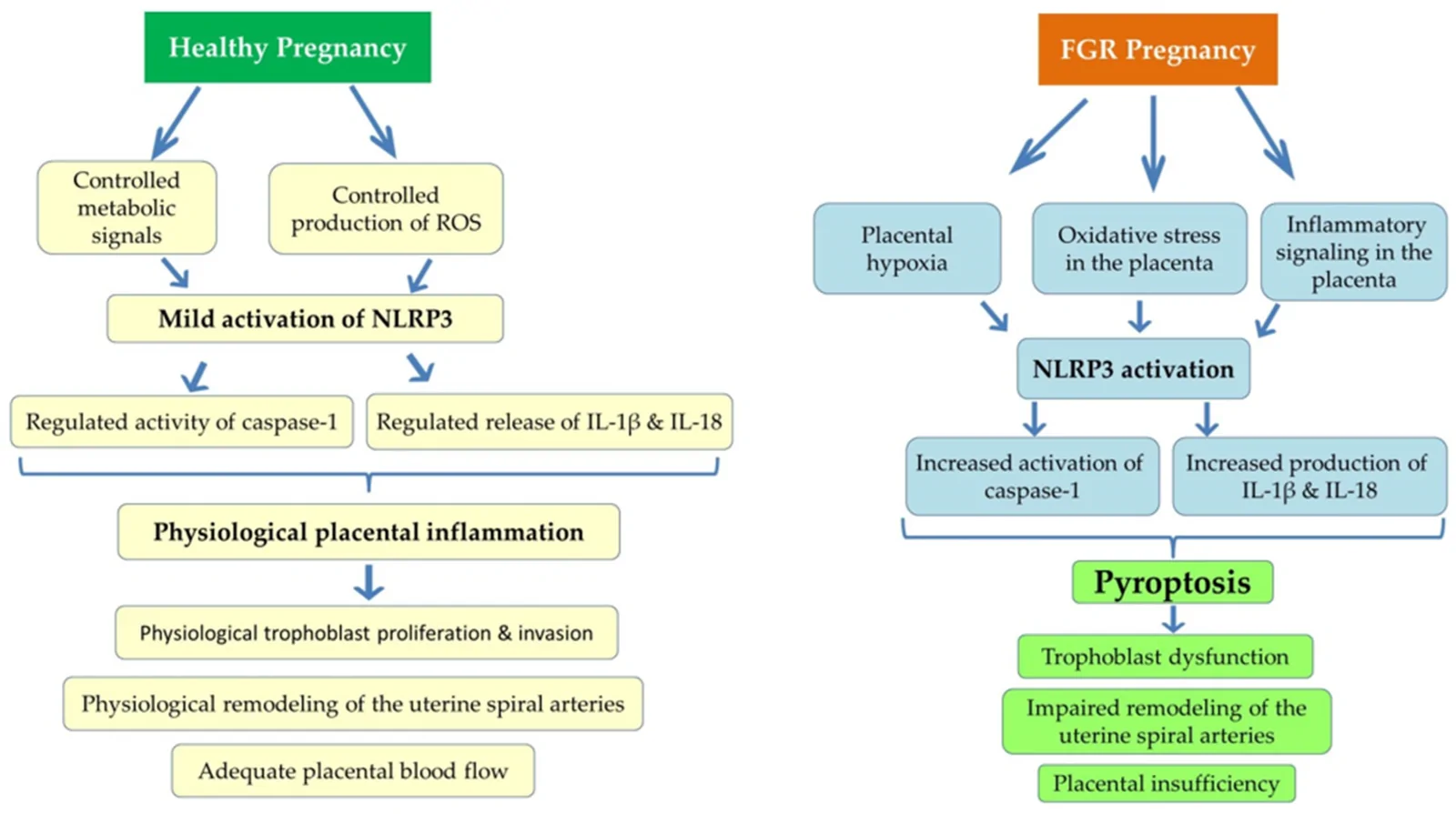
Role of the NLRP3 inflammasome in maintaining placental homeostasis during normal pregnancy and the contribution of its hyperactivation to inflammation, trophoblast pyroptosis, and the pathogenesis of FGR (abbreviations: NLRP3: NOD-like receptor family pyrin domain containing 3; IL-1β: Interleukin-1β; IL-18: Interleukin-18; FGR: Fetal growth restriction).

The NLRP3 inflammasome is an intracellular multiprotein complex of innate immunity that activates caspase-1 and promotes maturation of IL-1β and IL-18 [41,42]. In healthy pregnancy, controlled inflammasome activity contributes to local immune surveillance and placental adaptation, whereas excessive activation may disturb trophoblast and vascular function [43,44] (Figure 3).

In FGR, increased placental NLRP3 activation has been accompanied by higher caspase-1, IL-1β and IL-18 expression. Activation of the NLRP3/caspase-1 axis can promote gasdermin-D-dependent pyroptosis and release of inflammatory mediators, thereby impairing trophoblast proliferation, migration and invasion. These changes may interfere with spiral-artery remodeling and placental vascular development and contribute to placental insufficiency [45,46] (Figure 3).

Overall, current evidence supports NLRP3 inflammasome activation as an important inflammatory mechanism in FGR. Its association with altered cytokine production and trophoblast cell death identifies this pathway as a potential biomarker and experimental therapeutic target, although its diagnostic value and clinical safety require further validation.

### 3.4. Inflammation-Mediated Placental Remodeling and MMP-9 Signaling

MMP-9 is a key proteolytic enzyme involved in extracellular matrix (ECM) degradation, trophoblast invasion, angiogenesis, and placental tissue remodeling during normal pregnancy [47]. Expressed by trophoblasts, MMP-9 facilitates extravillous trophoblast invasion by degrading basement membrane components, including type IV collagen and laminin, thereby contributing to spiral artery remodeling and the establishment of adequate uteroplacental perfusion [48,49,50]. Through its effects on trophoblast migration, differentiation, and vascular remodeling, MMP-9 contributes to the development of a functional placental vascular network essential for adequate fetal oxygen and nutrient supply [51].

In FGR, placental MMP-9 expression and activity appear to be dysregulated and have been associated with impaired trophoblast invasion, incomplete spiral-artery remodeling and altered placental vascularization [50,52,53]. Altered MMP-9/TIMP-1 balance may influence extracellular matrix turnover and the local availability of cytokines and growth factors. Because MMP-9 activity varies according to tissue compartment, gestational age and disease phenotype, its contribution should be interpreted within the broader process of placental remodeling rather than as an isolated biomarker.

Hypoxia and inflammatory signaling may further modify MMP-9 regulation through NF-κB, AP-1 and HIF-dependent pathways. The resulting imbalance in extracellular matrix turnover may impair trophoblast invasion, spiral-artery remodeling and development of the fetoplacental vascular network [50].

MMP-9 also plays an important role in angiogenic remodeling by modifying the ECM and increasing the bioavailability of matrix-sequestered proangiogenic factors, particularly VEGF [54]. Dysregulated MMP-9 activity may therefore contribute to alterations in the local angiogenic environment and the imbalance between pro- and antiangiogenic factors characteristic of placental dysfunction. Such changes may impair placental vascular development and ultimately compromise fetal oxygen and nutrient exchange [55,56,57]. The biological effects of MMP-9 are further determined by its balance with tissue inhibitors of metalloproteinases (TIMPs), particularly TIMP-1. In human FGR placentas, reduced MMP-9 expression together with increased TIMP-1 and TIMP-3 expression has been reported, suggesting altered proteolytic balance and impaired ECM remodeling [58,59].

Overall, dysregulation of the MMP-9/TIMP system in FGR may represent a convergence point linking ECM remodeling, trophoblast invasion, inflammation, hypoxia, and angiogenesis. Rather than acting as an isolated mediator, MMP-9 may participate in a broader pathological network that compromises placental structure and function, thereby contributing to FGR. These interconnected pathways also highlight MMP-9 and its regulatory mechanisms as potential targets for further investigation in FGR.

## 4. The Role of Growth-Factor and Angiogenic Dysregulation in the Pathophysiology of FGR

### 4.1. Placental Growth Factor (PlGF) as a Key Regulator of Healthy and FGR Pregnancies

Placental Growth Factor (PlGF) is an important member of the vascular endothelial growth factor (VEGF) family and actively participates in the regulation of angiogenesis in the human placenta through its binding to the VEGFR-1 receptor, for which it displays high affinity [60,61]. Its production occurs mainly by the syncytiotrophoblast and its secretion into the maternal circulation makes PlGF an important factor for the normal development of pregnancy [62].

**Figure 4:**
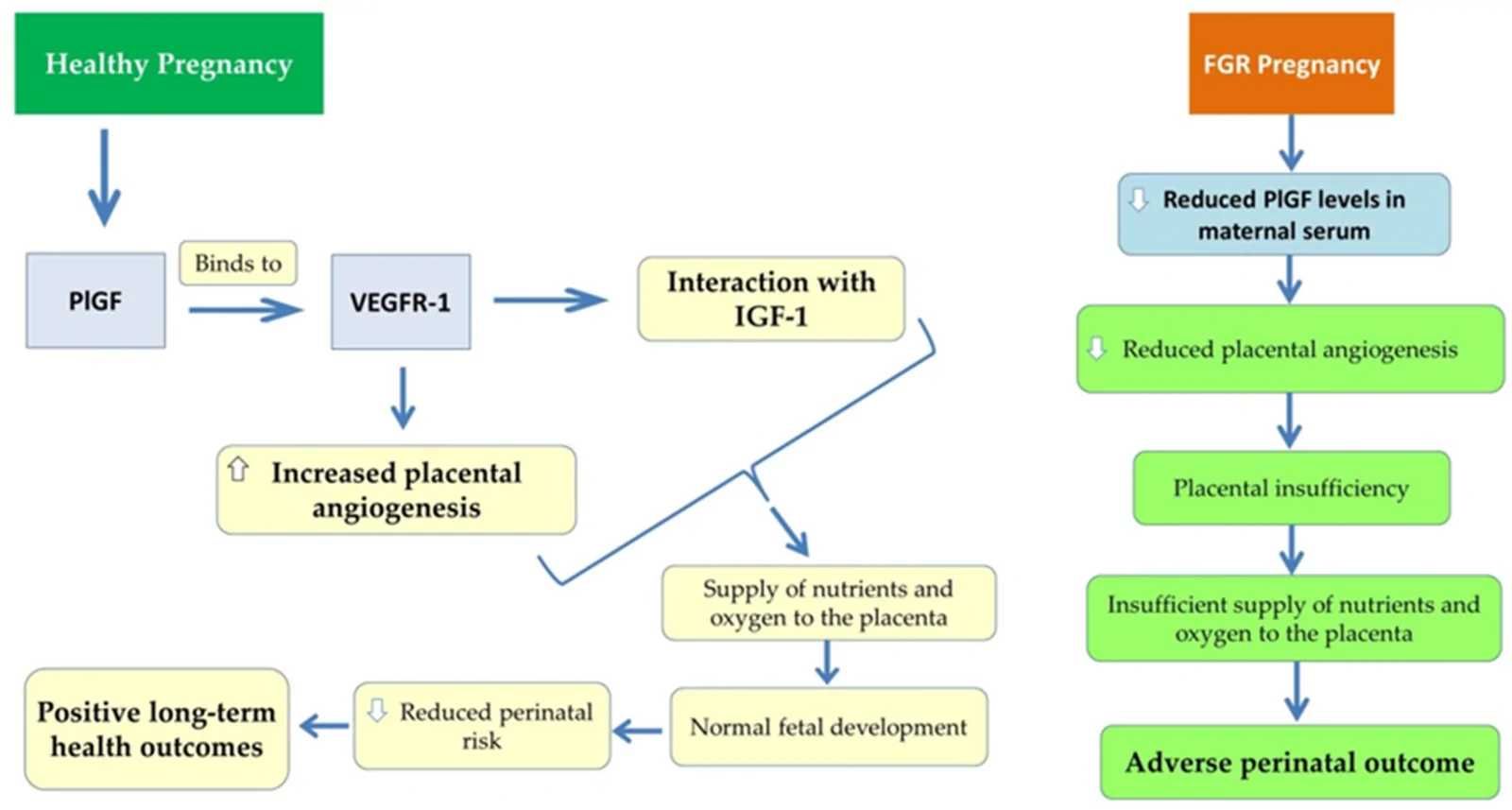
Schematic illustration of the role of PlGF in healthy pregnancies and pregnancies complicated by FGR (abbreviations: PIGF: Placental growth factor; VEGFR-1: Vascular endothelial growth factor receptor 1; IGF-1: Insulin-like growth factor 1; FGR: Fetal growth restriction).

PlGF contributes to the maintenance of pregnancy, regulating the distribution of nutrients between the mother and fetus. In particular, it affects both the availability of nutrients to the mother and their transport to the fetus, playing a crucial role in uteroplacental function [63]. At the same time, its action is linked to placental development, the normal adaptation of the maternal organism during pregnancy and fetal development through interactions with the insulin-like growth factor-I (IGF-I) signaling pathways and the regulation of maternal metabolism [64,65] (Figure 4).

Reduced concentrations of PlGF in the maternal circulation have been associated with FGR. One possible mechanism involves dysfunction of the growth hormone/insulin-like growth factor-I (GH/IGF-I) axis, which may limit fetal growth and contribute to the occurrence of FGR pregnancy [64,65]. Furthermore, it has been reported that FGR pregnancies exhibit significantly lower levels of PlGF in the maternal circulation compared to healthy pregnancies [66] (Figure 4).

The study by Hong and Kumar suggests that PlGF is one of the most promising circulating markers of placental dysfunction, with potential value in the early prediction and recognition of FGR [67]. Although current evidence supports its potential utility as a biomarker of placental function, further research is needed to fully elucidate the molecular mechanisms through which PlGF influences fetal development and to establish its clinical applicability in the prediction and management of FGR.

### 4.2. VEGF Signaling in Placental Vascular Development and Angiogenic Dysfunction

Vascular endothelial growth factor is one of the most important regulators of angiogenesis in the placenta. Its biological action is exerted mainly through its binding to the VEGFR-1 (Flt-1) and VEGFR-2 (KDR) receptors, which activate molecular pathways that promote the proliferation, migration, survival and remodeling of endothelial cells, contributing to the creation and maintenance of a functional vascular network in the placenta [68,69]. Normal VEGF signaling is essential for adequate placental vascularization, while disturbances in its expression or activity have been associated with placental insufficiency and FGR [70].

**Figure 5:**
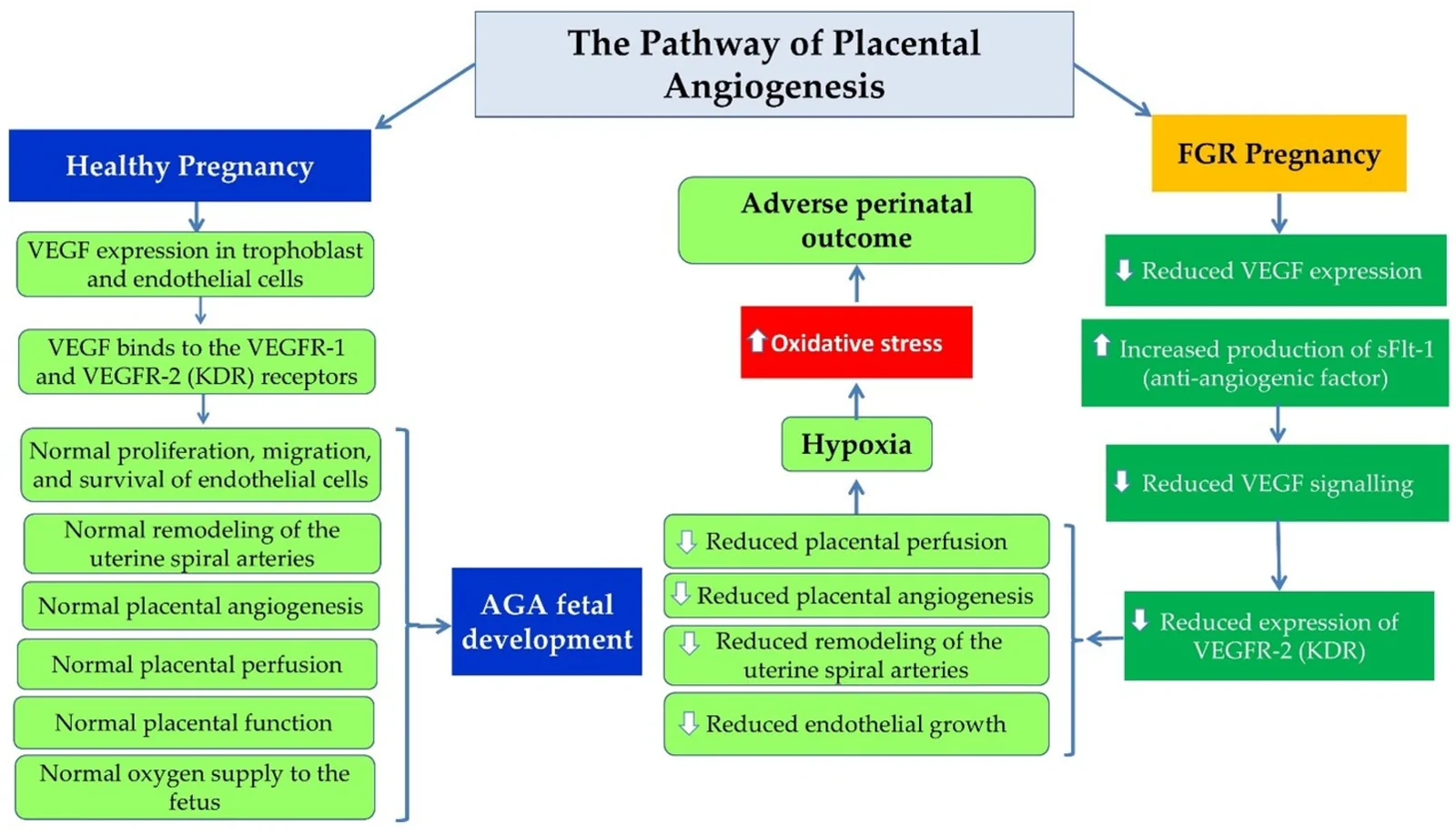
Schematic illustration of the role of VEGF in healthy pregnancies and pregnancies complicated by FGR (abbreviations: VEGF: Vascular endothelial growth factor; VEGFR-1: Vascular endothelial growth factor receptor 1; VEGFR-2: Vascular endothelial growth factor receptor 2; sFlt-1: Soluble fms-like tyrosine kinase-1; AGA: Appropriate for Gestational Age; FGR: Fetal Growth Restriction).

During healthy pregnancy, VEGF is expressed in various cell populations of the placenta, including cytotrophoblasts, syncytiotrophoblasts, and placental vascular endothelial cells. The wide distribution of both VEGF and its receptors suggests that this factor exerts autocrine and paracrine actions, which are essential for the normal function of trophoblast cells and the development of the placental vascular network [69,71] (Figure 5).

In pregnancies complicated by FGR, impaired VEGF signaling and reduced expression of its receptors, particularly VEGFR-2 (KDR), have been observed, reflecting impaired angiogenic activity and inadequate development of the placental vascular network [72]. Furthermore, gene expression studies have revealed significant alterations in genes and microRNAs involved in the regulation of angiogenesis in the placentas of FGR, suggesting that disrupted angiogenic pathways contribute to the pathological formation of placental vascular architecture and the reduction in blood flow to the fetus [73] (Figure 5).

Hypoxia, which is a hallmark of placental insufficiency, also affects the regulation of VEGF and related angiogenic factors [74]. Under conditions of low oxygenation, the balance between proangiogenic and antiangiogenic factors is altered, leading to further deterioration of placental function [75]. At the same time, placentas associated with FGR exhibit reduced expression of VEGF in combination with increased levels of the antiangiogenic factor sFlt-1 and increased markers, such as malondialdehyde (MDA) and 8-hydroxy-2΄-deoxyguanosine (8-OHdG) [76]. These findings support the existence of a close interaction between angiogenic imbalance and hypoxia in the pathogenesis of FGR.

Overall, VEGF is a fundamental regulator of placental angiogenesis and AGA fetal development. Reduced VEGF activity, impaired expression of its receptors, disruption of angiogenic gene networks, and hypoxia-induced effects contribute to placental dysfunction and reduced uteroplacental perfusion. Accordingly, impaired VEGF signaling is considered one of the key mechanisms linking placental insufficiency to FGR. A deeper understanding of these molecular mechanisms may facilitate the identification of novel diagnostic biomarkers and the development of therapeutic approaches aimed at improving placental function and fetal outcomes in high-risk pregnancies.

### 4.3. The TGF-α/EGFR Signaling Pathway in Placental Development and FGR

TGF-α is a member of the epidermal growth factor (EGF) family, which is involved in the regulation of cell proliferation, differentiation and angiogenesis [77]. Current research data indicate that disruption of TGF-α expression and action in the placenta may contribute to placental dysfunction and, by extension, to FGR [78]. During healthy pregnancy, TGF-α is expressed in placental trophoblast cells and it has been shown that its exogenous administration stimulates their proliferation, thus enhancing placental growth and maturation [79]. Its biological action is exerted mainly through interaction with the epidermal growth factor receptor (EGFR), activating signaling pathways that regulate the proliferation, differentiation, invasion, and fusion of trophoblast cells. These processes are essential for the proper formation of the placenta and the remodeling of its vascular network [78] (Figure 5).

In pregnancies complicated by FGR, increased concentrations of TGF-α have been observed in cord blood compared with AGA neonates. These findings suggest alterations in the regulation of this growth factor and possible dysfunction of placental growth mechanisms [80]. At the same time, the expression of TGF-α, EGF and other members of the EGF family of growth factors appears reduced in the placental tissue of pregnancies with FGR, which supports the view that the attenuation of the relevant signaling contributes to the occurrence of placental insufficiency [78]. Insufficient activation of these pathways may negatively affect the functionality of trophoblast cells and limit normal vascular development of the placenta (Figure 5).

**Figure 6:**
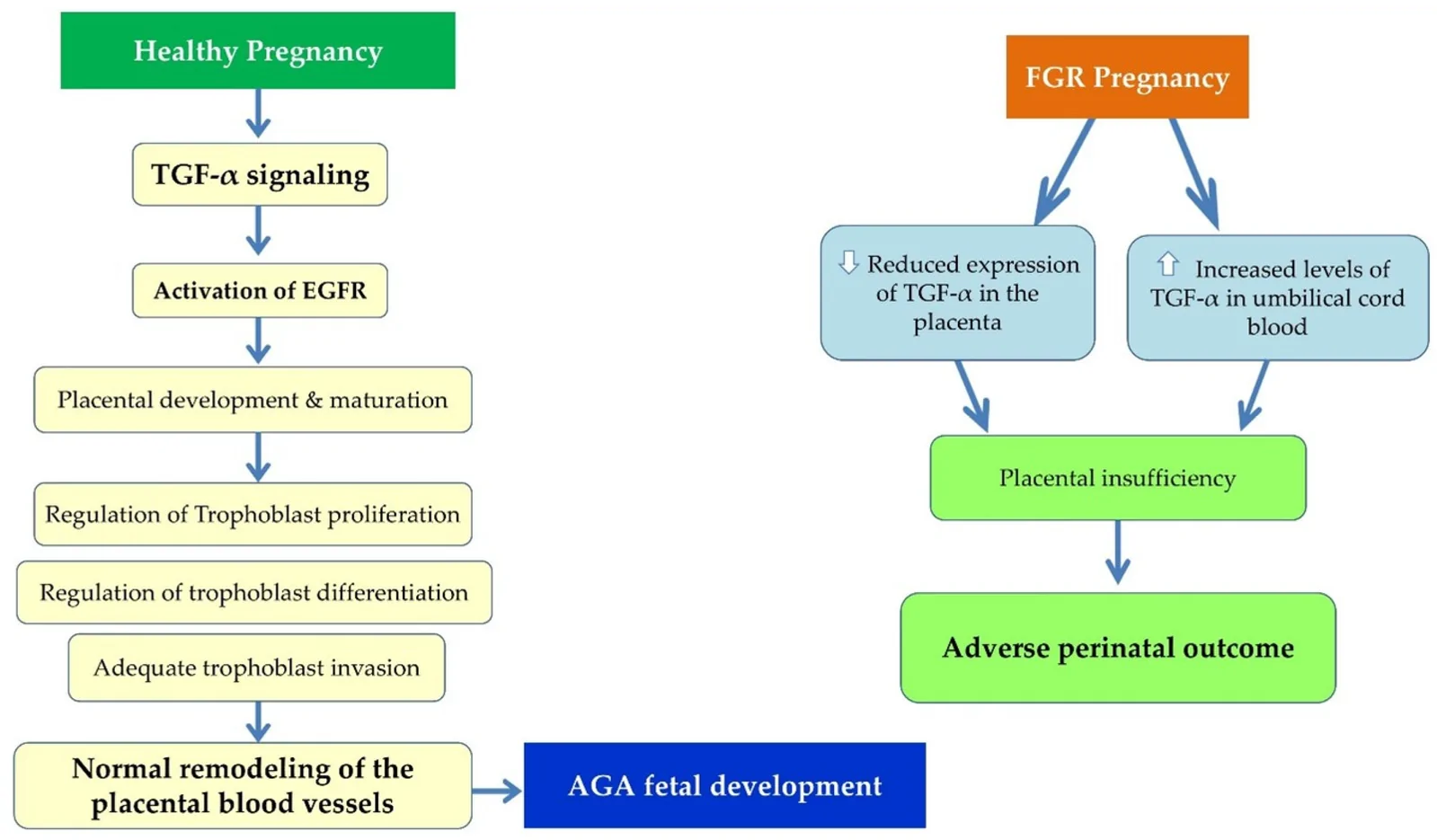
Schematic illustration of the role of TGF-α in healthy pregnancies and pregnancies complicated by FGR (abbreviations: TGF-α: Transforming Growth Factor-alpha; EGFR: Epidermal Growth Factor Receptor; AGA: Appropriate for Gestational Age; FGR: Fetal Growth Restriction).

Overall, TGF-α plays a crucial role in normal placental development and function by regulating trophoblast proliferation, differentiation, and invasive capacity, as well as vascular remodeling through EGFR activation. In pregnancies complicated by FGR, dysregulation of the TGF-α/EGFR pathway has been observed, characterized by increased TGF-α levels in the fetal circulation but reduced placental expression. These alterations may contribute to trophoblast dysfunction, impaired angiogenesis, and the development of placental insufficiency, ultimately leading to FGR. A deeper understanding of the role of TGF-α in placental biology and the molecular mechanisms underlying its dysregulation in FGR may facilitate the identification of novel biomarkers and the development of targeted therapeutic interventions aimed at improving fetal outcomes in high-risk pregnancies(Figure 6).

### 4.4. The Role of Transforming Growth Factor-Beta 2 (TGF-β2) in Placental Development and FGR

Transforming growth factor-beta 2 (TGF-β2) is a multifunctional cytokine and growth factor belonging to the TGF-β superfamily that contributes to cell differentiation, immunomodulation, extracellular matrix remodeling, and placental development [81]. In the human placenta, TGF-β2 is expressed at the maternal–fetal interface and is involved in the regulation of trophoblast function, including proliferation, differentiation, and invasion [81,82]. TGF-β2 expression in the human placenta appears to be the predominant TGF-β isoform during the third trimester, with expression localized to trophoblast populations, the chorionic plate, the decidua, and the placental basal plate. Its tightly regulated signaling may therefore contribute to placental homeostasis and trophoblast function [81]. During early human placentation, TGF-β2 negatively regulates extravillous trophoblast (EVT) invasion and outgrowth and contributes to the regulation of endovascular EVT differentiation and spiral artery remodeling [83,84,85]. In parallel, TGF-β2 may contribute to maternal–fetal immune tolerance by participating in the immunoregulatory milieu at the maternal–fetal interface, while TGF-β signaling more broadly promotes regulatory T-cell differentiation and modulates uterine/decidual natural killer-cell function [81,86,87]. Also, TGF-β2 is involved in regulating trophoblast function, placental vascular development, angiogenesis, and extracellular matrix remodeling through SMAD-dependent and non-canonical pathways, while interacting with broader angiogenic and matrix-remodeling mechanisms that contribute to placental function [85,87,88].

Dysregulated TGF-β/SMAD2/3 signaling may impair trophoblast function and contribute to FGR [87,89,90]. Altered TGF-β2 signaling may also disturb extracellular matrix remodeling within the placenta, potentially affecting trophoblast differentiation, vascular development and tissue architecture [81].

Overall, TGF-β2 emerges as an important component of the regulatory network governing trophoblast function, maternal–fetal immune homeostasis, extracellular matrix remodeling, and placental vascular adaptation. In pregnancies complicated by FGR, the hypoxic and inflammatory placental environment may alter TGF-β signaling and its downstream SMAD-dependent pathways, potentially disrupting the coordinated regulation of trophoblast invasion, extracellular matrix remodeling, and vascular function. Such dysregulation may contribute to abnormal placental architecture, endothelial dysfunction, and impaired placental homeostasis, thereby providing a potential mechanistic link between altered TGF-β signaling and FGR.

### 4.5. The Insulin-like Growth Factor (IGF) System in Placental Development and FGR

AGA fetal development is largely dependent on the IGF system, which comprises IGF-I and IGF-II, their receptors (primarily IGF-IR and IGF-IIR), and six insulin-like growth factor-binding proteins (IGFBP-1 to IGFBP-6) [91]. This system is a major regulator of placental and fetal development and controls critical cellular processes, including proliferation, differentiation, survival, apoptosis, and angiogenesis [92,93]. IGFBPs regulate the bioavailability, distribution, and activity of IGFs and can also exert IGF-independent effects that influence cellular growth, inflammation, and cellular stress responses [94].

In the normal placenta, both IGF-I and IGF-II are expressed and contribute to placental growth, trophoblast function, and maintenance of placental tissue homeostasis [92]. IGF signaling is important for trophoblast proliferation and survival, placental vascular development, and the regulation of nutrient and oxygen supply to the fetus [92,95]. In parallel, the regulated expression and activity of IGFBPs contribute to the control of local IGF bioavailability and to placental homeostasis and fetal growth [96].

Increasing evidence indicates that dysregulation of the IGF system is involved in the pathogenesis of FGR [97]. In pregnancies complicated by FGR, reduced IGF-I expression and/or signaling has been reported in placental tissue and fetal circulation, and lower fetal IGF-I concentrations are associated with reduced birth weight [98,99]. In contrast, IGF-II expression and signaling appear to be more complex and may vary according to placental tissue, gestational age, and the underlying cause of FGR; therefore, altered rather than uniformly reduced IGF-II signaling is a more appropriate description [92,99,100]. Experimental and human studies indicate that impaired IGF signaling may affect trophoblast proliferation and survival, placental angiogenesis, and nutrient transport, thereby contributing to placental insufficiency and FGR [92,100,101].

Recent evidence has further highlighted the potential involvement of specific Insulin-like Growth Factor-I (IGF-I) splice isoforms, particularly IGF-IEa and IGF-IEb, in the placental dysfunction associated with FGR. Interestingly, both isoforms share a common transcriptional profile: recent studies by Fasoulopoulos et al. demonstrated that neither total placental IGF-IEa (2025) [102] nor IGF-IEb (2026) [103] mRNA levels differ significantly between FGR and AGA pregnancies. However, at the protein level, they undergo highly dynamic, isoform- and compartment-specific regulation, exhibiting distinct immunoreactivity patterns that reflect different underlying pathological or adaptive mechanisms [102,103].

Specifically, protein immunoreactivity for the IGF-IEa isoform is frequently reduced or entirely absent in the extravillous trophoblasts of FGR placentas. This localized loss correlates with histological features of maternal vascular malperfusion and suggests a direct association with defective placentation [102]. Conversely, the IGF-IEb isoform exhibits increased protein expression in the perivillous syncytiotrophoblast, which correlates with maternal vascular malperfusion and specific clinical parameters, including gestational age, neonatal birth weight, placental weight, and fetal sex [103]. Despite these contrasting expression patterns across trophoblast compartments, both isoforms exhibit increased immunoreactivity in the endothelium of fetal villous and maternal decidual vessels in FGR [102,103]. This vascular upregulation may reflect a shared, localized adaptive response to placental vascular insufficiency and hypoxia, potentially aimed at promoting angiogenesis and vasodilation to compensate for impaired perfusion.

Importantly, the marked discrepancy between unchanged mRNA expression and altered protein distribution strongly suggests the involvement of complex post-transcriptional regulatory mechanisms governing both isoforms in the context of altered local signaling [102,103]. Nevertheless, the available evidence remains strictly observational and does not establish a causal relationship or direct functional role for either IGF-IEa or IGF-IEb in the development of FGR. Further mechanistic, functional, and prospective studies are therefore needed to determine whether these isoforms represent clinically useful biomarkers of placental dysfunction or active molecular mediators of placental tissue adaptation [102,103].

Increased circulating IGFBP-1 concentrations and enhanced site-specific phosphorylation of IGFBP-1 have been reported in pregnancies complicated by FGR, particularly in fetal and umbilical cord blood. Increased phosphorylation of IGFBP-1 at specific serine residues, including Ser101, Ser119, and Ser169, increases its affinity for IGF-I and may promote IGF-I sequestration, thereby reducing IGF-I bioavailability and IGF-I receptor signaling [104,105]. Experimental studies further indicate that hypoxia and nutrient deprivation can promote IGFBP-1 hyperphosphorylation, providing a potential mechanistic link between the adverse intrauterine environment, altered IGF-I bioavailability, and FGR [105,106]. In addition, altered expression and proteolytic processing of IGFBPs may modify local IGF bioavailability at the maternal–fetal interface and contribute to abnormal placental function [98]. IGFBP-4 and IGFBP-5 are expressed in the developing placenta and can modulate IGF-dependent trophoblast migration and invasion. Experimental evidence indicates that IGFBP-4 and IGFBP-5 may also exert IGF-independent effects on placental cellular processes, although their specific contribution to human FGR remains incompletely defined [107,108]. The IGF/IGFBP system is closely integrated with inflammatory pathways, which influence IGF bioavailability and trophoblast function in FGR [109]. Increased IL-6 concentrations have been reported alongside altered IGFBP-1 and IGFBP-2 levels, suggesting that inflammatory signaling may modify growth-factor activity within the placenta [99].

Overall, the IGF/IGFBP system plays a central role in regulating placental function and fetal development. In pregnancies complicated by FGR, dysregulation of IGF-I, IGF-II, and IGFBPs is closely associated with alterations in trophoblast function, placental development, and inflammatory processes, potentially contributing to placental insufficiency and impaired fetal growth. Further characterization of these molecular interactions may facilitate the identification of novel biomarkers of placental dysfunction and improve our understanding of potential therapeutic targets for FGR.

### 4.6. Angiogenic Imbalance and the Role of sFlt-1 in FGR

Disturbed placentation and dysfunction of the placental vascular network are key pathophysiological mechanisms of FGR [1,110,111,112]. One of the most important antiangiogenic factors associated with placental insufficiency is the sFlt-1, which is considered an indicator of a disruption in the balance between angiogenic and antiangiogenic factors in the placenta [113]. sFlt-1 functions as a circulating decoy receptor for VEGF and PlGF, binding to these factors and thereby reducing their bioavailability. As a result, the normal development of the placental vascular network is restricted and angiogenesis is disrupted, which contributes to the occurrence of placental dysfunction [114].

In pregnancies complicated by FGR, increased values of the sFlt-1/PlGF ratio in maternal serum have been recorded, a finding that reflects the presence of placental insufficiency. Systematic analyses including more than 25,000 pregnancies have shown that women with FGR consistently present higher values of the sFlt-1/PlGF ratio compared to pregnancies with AGA growing fetuses [113,115,116]. A similar increase is observed in preeclampsia, although the levels of the sFlt-1/PlGF ratio are usually higher in preeclampsia than in FGR, suggesting that the two conditions share a common background of angiogenic dysregulation but with different severity [113,115,116]. Furthermore, elevated levels of sFlt-1 and the sFlt-1/PlGF ratio are associated with reduced concentrations of free VEGF and PlGF, endothelial dysfunction, and reduced uteroplacental perfusion [113]. In contrast, in healthy pregnancies, a gradual increase in sFlt-1 levels is observed during the third trimester, which is considered part of the normal regulation of angiogenesis and does not negatively affect fetal development [117].

Overall, elevated sFlt-1 levels in maternal circulation and placental tissue in pregnancies complicated by FGR reflect an impaired placental angiogenic balance and contribute to endothelial dysfunction and reduced uteroplacental perfusion. These findings suggest that sFlt-1 is not only a key mediator of placental insufficiency but also a promising biomarker for assessing angiogenic dysregulation in FGR, distinguishing pathological alterations from the physiological changes occurring during normal pregnancy.

## 5. Tumor Suppressor and Chromatin-Regulatory Pathways in FGR

**Figure 7:**
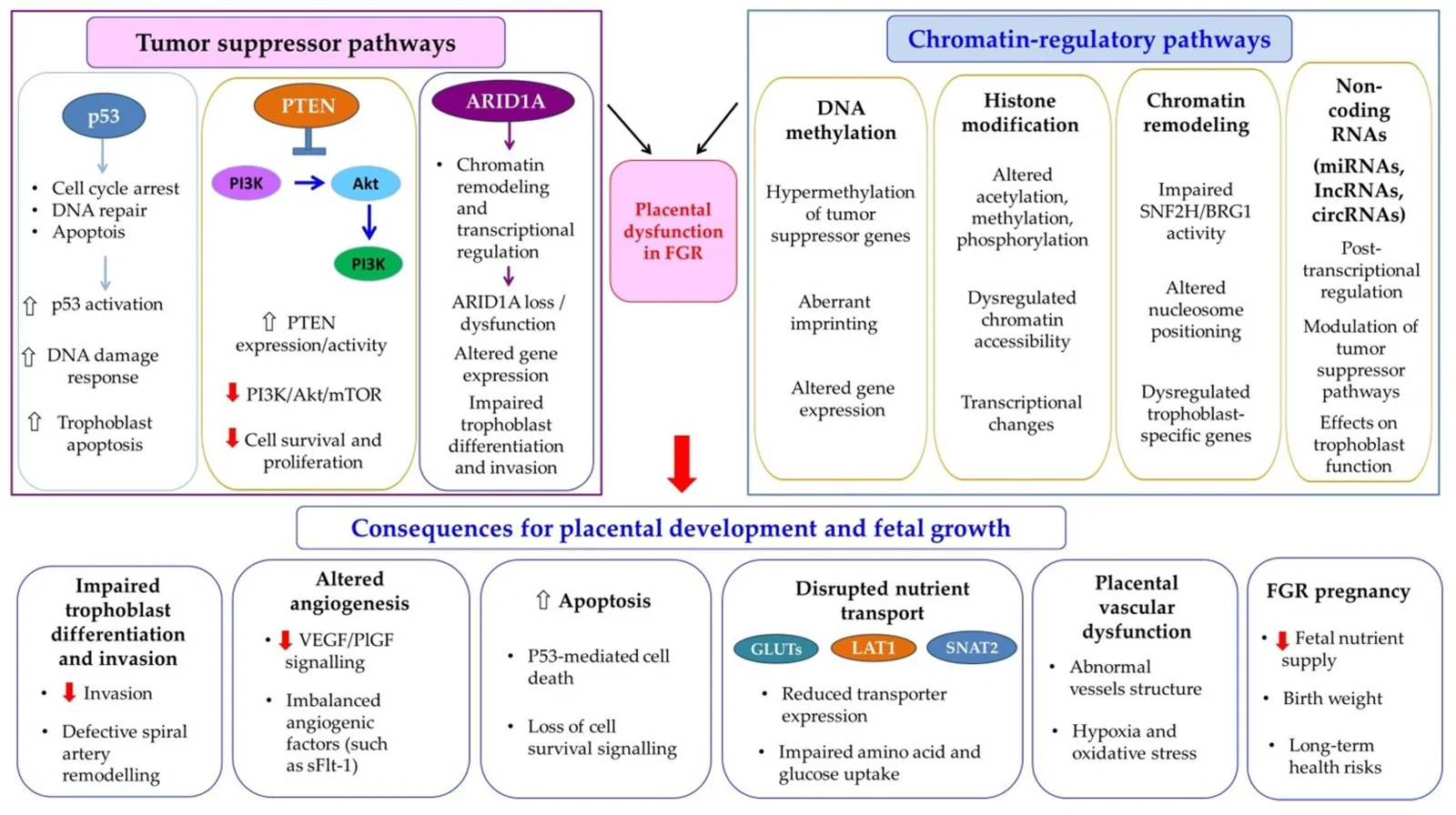
Tumor suppressor and chromatin-regulatory pathways in FGR. Schematic illustration of the interconnected molecular networks governing placental development and fetal growth. The left panel outlines the pathological hyperactivation or dysfunction of key tumor suppressor pathways, whereby p53 induction promotes cell-cycle arrest and apoptosis, PTEN upregulation inhibits downstream PI3K/Akt/mTOR signaling, thereby reducing cell survival, and ARID1A loss disrupts trophoblast differentiation and migratory capacity. The right panel illustrates the corresponding layers of chromatin-regulatory mechanisms, including promoter hypermethylation, altered histone acetylation and methylation, impaired SWI/SNF (SNF2H/BRG1)-mediated nucleosome positioning, and dysregulated post-transcriptional microRNA/lncRNA networks. The convergence of these upstream epigenetic and genomic alterations compromises the maternal–fetal interface, leading to impaired trophoblast invasion, defective angiogenesis, increased cellular apoptosis, and impaired nutrient transporter trafficking, ultimately resulting in placental vascular dysfunction and FGR.

Placental development and structural homeostasis require strict, multidimensional coordination between intrinsic gene transcription networks, cell-cycle checkpoints, and cell survival signaling pathways at the maternal–fetal interface. While physiological early-stage placentation depends on the synchronized invasion of extravillous trophoblasts (EVTs) and rapid villous tissue proliferation, the severe ischemic and hypoxic stress conditions characterizing pathological placental insufficiency can permanently disrupt this biological equilibrium. At the direct core of this structural breakdown lies a sophisticated regulatory cross-talk handled by coordinated tumor suppressor pathways and upstream chromatin-regulatory mechanisms. As systematically illustrated in Figure 7, the orchestration of healthy placental development is tightly governed by two primary intrinsic molecular axes: tumor suppressor genes, including p53, PTEN, and ARID1A, acting as master cellular quality-control hubs; and detailed epigenetic modifications, encompassing global DNA methylation patterns, post-translational histone modifications, ATP-dependent chromatin remodeling complex dynamics, and non-coding RNA networks.

When these protective molecular pathways are compromised, the cellular architecture of the placenta undergoes profound and self-reinforcing pathological changes. Rather than maintaining normal tissue homeostasis and turnover, aberrant signaling shifts trophoblast differentiation toward structural deterioration, chronic cell-cycle arrest, and accelerated cellular senescence. The consequences of this molecular disruption extend beyond the placenta, directly and adversely affecting the developing fetal compartment. As illustrated by the functional hierarchy in Figure 7, the combined effects of tumor suppressor pathway hyperactivation and epigenetically mediated chromatin silencing give rise to a unified pathogenic phenotype. This disruption of molecular networks is manifested clinically by impaired EVT invasion, defective spiral artery remodeling, and profound angiogenic imbalance, characterized by reduced VEGF/PlGF signaling and increased production of the anti-angiogenic factor sFlt-1. At the cellular level, these alterations promote p53-mediated cell death, impair PI3K/Akt-dependent survival signaling, and disrupt the expression and trafficking of key glucose and essential amino acid transporters, particularly GLUT1, SNAT2, and LAT1. Consequently, this multilayered pathological network contributes to placental vascular dysfunction, compromising the oxygen and nutrient supply required for normal embryonic and fetal development and ultimately restricting fetal growth potential.

### 5.1. The Role of the p53 Pathway in Trophoblast Apoptosis and FGR

The tumor suppressor p53 is a central regulator of cell-cycle arrest, DNA repair, apoptosis and senescence. During normal placental development, tightly controlled p53 activity helps maintain trophoblast homeostasis by balancing proliferation with removal of damaged or excess cells [1,118].

In pregnancies complicated by FGR, cellular stress may trigger p53 activation promoting trophoblast apoptosis and contributing to placental dysfunction [1,119]. Research data have demonstrated increased p53 expression and activation in placentas from FGR pregnancies, particularly within the trophoblast compartment, while experimental evidence further implicates p53 signaling in trophoblast differentiation and placental vascular abnormalities [1]. Increased p53 activity can induce cell-cycle arrest and restrict trophoblast proliferation, while also activating pro-apoptotic pathways that promote trophoblast cell death [120,121,122]. Excessive loss of trophoblast cells may reduce the functional surface of the placenta and negatively affect trophoblast renewal and differentiation [56]. In parallel, dysregulated p53 activity may contribute to impaired placental vascular development and angiogenesis, potentially exacerbating the reduced vascular network characteristic of FGR and limiting the capacity of the placenta to meet the increasing oxygen and nutrient demands of the developing fetus [56]. p53 signaling may also respond to inflammatory and cellular stress associated with placental insufficiency. Placental dysfunction can promote p53 activation, cell-cycle arrest and apoptosis, providing a potential explanation for increased trophoblast cell death in FGR [1].

Overall, p53 appears to be an important regulator of trophoblast fate in FGR. Dysregulation of p53 signaling may promote cell-cycle arrest, apoptosis, and cellular senescence, while also influencing placental vascular development. These findings support further investigation of p53-related pathways as potential biomarkers of placental dysfunction, although their clinical utility remains to be established.

### 5.2. PTEN-Dependent Regulation of Placental Cell Survival and Angiogenic Signaling in FGR

Phosphatase and Tensin Homolog (PTEN) is a lipid phosphatase that acts as a major negative regulator of the phosphoinositide 3-kinase/protein kinase B (PI3K/AKT) signaling pathway. By dephosphorylating phosphatidylinositol-3,4,5-trisphosphate (PIP3) to phosphatidylinositol-4,5-bisphosphate (PIP2), PTEN limits PIP3-dependent AKT activation and thereby modulates downstream cellular responses. In the placenta, PI3K/AKT signaling regulates fundamental processes essential for placental development and function, including trophoblast proliferation, survival, differentiation, metabolism, and vascularization. Notably, impaired PI3K/AKT signaling has been reported in FGR placentas, suggesting that dysregulation of this pathway may contribute to placental dysfunction and FGR [123,124]. In placental tissues, the balance between PI3K/AKT activation and PTEN-mediated inhibition is therefore important for maintaining trophoblast function and normal placental development. Experimental evidence indicates that PTEN contributes to the regulation of trophoblast invasion and placental vascular remodeling, while alterations in PI3K/AKT signaling have been associated with impaired placental function and fetal growth [123,124].

In pregnancies complicated by FGR, evidence suggests that dysregulation of the PTEN/PI3K/AKT axis may contribute to impaired placental function. However, direct evidence that PTEN expression is consistently increased in FGR placental tissue remains limited. Studies of FGR placentas have identified alterations in regulatory miRNAs targeting PTEN, consistent with enhanced PTEN-mediated inhibition of PI3K/AKT signaling, while experimental studies demonstrate that PTEN can regulate trophoblast migration and invasion and influence placental and uterine vascular remodeling [123,125]. In addition, reduced PI3K and AKT phosphorylation has been demonstrated in human FGR placentas, providing evidence of impaired pathway activity in FGR [124]. Thus, dysregulation of PTEN or other components of the PI3K/AKT pathway may impair trophoblast proliferation, migration, survival, and vascular development, potentially contributing to placental insufficiency in FGR.

Inflammatory signaling may also influence PTEN/PI3K/AKT activity in trophoblasts. Experimental activation of inflammatory pathways has been shown to alter PTEN expression and trophoblast invasion, suggesting that PTEN may act as a signaling node connecting inflammatory cues with placental cell behavior [126]. Whether this mechanism is directly involved in human FGR remains to be determined and requires further study.

Overall, PTEN is an important regulator of placental development through its modulation of PI3K/AKT signaling. Although consistent alterations in PTEN expression have not yet been established in human FGR, dysregulation of the PTEN/PI3K/AKT axis may affect trophoblast proliferation, migration, invasion, and survival, as well as placental vascular development. Further studies are needed to clarify which components of this pathway are altered in specific FGR phenotypes and to determine their potential relevance as biomarkers or therapeutic targets.

### 5.3. ARID1A Regulation of Trophoblast Differentiation and Placental Function in FGR

AT-rich interactive domain-containing protein 1A (ARID1A) is a core regulatory subunit of the SWI/SNF (Switch/Sucrose Non-Fermentable) chromatin-remodeling complex and contributes to the regulation of chromatin accessibility and transcriptional activity. Through this epigenetic function, ARID1A participates in cellular differentiation, proliferation, survival, and adaptation to cellular stress, processes that are particularly relevant to placental development and trophoblast function. In the human placenta, ARID1A is expressed in trophoblast populations and displays a distinct spatial pattern during normal placental development. Fantone et al. (2020) demonstrated that ARID1A is predominantly expressed in villous and extravillous cytotrophoblasts in normal placenta, whereas its expression is limited in the syncytiotrophoblast [126]. In placentas from pregnancies complicated by preeclampsia and intrauterine growth restriction (IUGR), however, ARID1A expression was also detected in the syncytiotrophoblast and syncytial knots, leading the authors to suggest that increased ARID1A expression in these compartments may be associated with impaired trophoblast differentiation [126].

The potential functional relevance of ARID1A to trophoblast biology is further supported by experimental evidence showing that ARID1A regulates trophoblast cell behaviour. In human trophoblast-like JEG-3 cells, ARID1A has been shown to modulate cell migration and invasion, with ARID1A overexpression suppressing both processes, whereas ARID1A knockdown enhanced them. These effects were associated with the regulation of Snail transcription and MMP-9 protein stability, indicating that ARID1A can influence cellular mechanisms involved in trophoblast motility and invasion [127]. Although these functional experiments were not performed specifically in FGR, they provide a biological rationale for considering altered ARID1A activity as a potential regulator of trophoblast behaviour in placental disorders.

Evidence directly linking ARID1A to FGR remains limited but is emerging. Altered ARID1A localization has been observed in placentas affected by FGR, particularly within the syncytiotrophoblast and syncytial knots, supporting an association between ARID1A dysregulation and abnormal trophoblast differentiation in growth-restricted pregnancies [126]. In late FGR, ARID1A expression may also vary within the placental vascular compartment in association with chronic placental insufficiency, suggesting that ARID1A could contribute to placental adaptation to vascular stress. However, the available evidence does not yet establish whether these alterations are causally involved in the pathogenesis of FGR or represent a placental response to chronic insufficiency. This distinction is important because FGR is characterized by complex abnormalities in placental development, including altered villous architecture and fetal vascular development [128].

ARID1A may also link placental chromatin regulation with cellular responses to adverse metabolic conditions. In placentas from pregnancies complicated by gestational diabetes, increased ARID1A expression has been observed, while ARID1A overexpression in trophoblast cells exposed to high glucose promoted cell-cycle arrest and cellular senescence. Mechanistically, ARID1A recruited GATA2 to the CDKN1A promoter, resulting in increased p21 expression and suppression of trophoblast proliferation [129]. Although these findings were obtained in the context of gestational diabetes rather than FGR, they provide experimental evidence that altered ARID1A activity may influence trophoblast homeostasis under conditions of metabolic stress [129]. Additional experimental findings indicate that ARID1A can regulate trophoblast homeostasis under adverse cellular conditions. In pregnancies complicated by gestational diabetes, increased placental ARID1A expression has been associated with trophoblast cell-cycle arrest and cellular senescence under high-glucose conditions. Mechanistically, ARID1A overexpression promotes the recruitment of GATA2 to the CDKN1A promoter, thereby increasing p21 expression and contributing to trophoblast dysfunction [129].

Taken together, current evidence supports a role for ARID1A in regulating trophoblast differentiation and cellular behavior, although its specific contribution to FGR remains incompletely understood. In particular, further FGR-specific studies are needed to determine whether alterations in ARID1A-dependent chromatin remodeling directly contribute to the transcriptional dysregulation, vascular abnormalities, and trophoblast dysfunction characteristic of placental insufficiency.

## 6. Epigenetic and Genomic Regulation of Placental Function: From Healthy Pregnancy to FGR

Epigenetic and genomic regulation constitute a fundamental layer of control over placental development, differentiation, and functional adaptation throughout pregnancy. The placental epigenome is established and dynamically modified during development, integrating developmental signals with maternal, nutritional, metabolic, and environmental influences. DNA methylation, histone modifications, ATP-dependent chromatin remodeling, genomic imprinting, and non-coding RNAs regulate the transcriptional programs required for trophoblast proliferation and differentiation, placental vascular development, nutrient transport, and endocrine function. Thus, epigenetic regulation should not be viewed exclusively as a pathological mechanism; rather, it is an essential component of normal placental development whose disruption may contribute to placental dysfunction and fetal growth restriction (FGR) [130,131,132].

In healthy pregnancy, epigenetic regulation permits cell-type-specific and gestational-age-dependent changes in gene expression while maintaining the functional plasticity of the placenta. Placental DNA methylation patterns differ from those of many somatic tissues, and both methylated and unmethylated genomic regions contribute to the regulation of trophoblast lineage specification and placental phenotype. Histone modifications and chromatin remodeling further determine the accessibility of regulatory DNA, whereas microRNAs (miRNAs) and other non-coding RNAs provide post-transcriptional control of gene networks. This multilayered regulation is particularly relevant to trophoblast invasion, differentiation, angiogenesis, and the capacity of the placenta to adapt to changing fetal and maternal demands [19,130].

Genomic imprinting represents a specialized epigenetic mechanism with particular relevance to placental biology. Imprinted genes are expressed according to parental origin and participate in the regulation of placental growth, morphology, and nutrient allocation. The IGF2/H19 locus is a well-characterized example. In normal development, the coordinated epigenetic regulation of this locus contributes to appropriate placental and fetal growth, while experimental disruption of placental Igf2 signaling can impair placental growth and subsequently fetal growth [101,131]. These findings support the concept that epigenetic regulation is an integral component of the mechanisms through which the placenta adapts its growth and transport capacity to meet fetal requirements.

### 6.1. DNA Methylation and Genomic Imprinting in Healthy Pregnancy and FGR

DNA methylation, predominantly occurring at CpG sites, is one of the principal mechanisms regulating gene transcription. In the placenta, methylation is developmentally dynamic and can influence promoters, enhancers, and other regulatory regions. During normal pregnancy, appropriate methylation patterns contribute to the establishment of trophoblast identity and the regulation of genes involved in placental growth, vascular development, endocrine activity, and nutrient exchange. Consequently, placental DNA methylation may reflect both intrinsic developmental programs and responses to the intrauterine environment [130,132].

Evidence from human placentas indicates that altered methylation of growth-related genes is associated with fetal growth phenotypes. Placental DNA methylation at IGF2, AHRR, HSD11B2, and WNT2 was associated with measures of fetal growth. Lower IGF2 methylation and higher HSD11B2 and WNT2 methylation were associated with increased odds of FGR. These findings support a relationship between placental DNA methylation and fetal growth; however, given the observational nature of such studies, they do not establish whether the observed methylation differences are causal, compensatory, or secondary to alterations in placental physiology [133].

Genome-wide approaches have extended these observations beyond individual candidate genes, providing evidence for broader epigenetic alterations in FGR. Differential DNA methylation has been reported in genomic regions involving genes and biological pathways related to angiogenesis, morphogenesis, immune regulation, and transcriptional control. Collectively, these findings suggest that altered DNA methylation may affect multiple biological processes involved in placental insufficiency rather than acting through a single growth-related gene. However, the interpretation of epigenome-wide associations in FGR should take into account differences in gestational age, placental cell composition, FGR phenotype, and analytical methodology, as these factors may contribute to variability between studies [134].

Genomic imprinting provides an additional mechanistic link between epigenetic regulation and fetal growth. Alterations in the H19/IGF2 region have been reported in placentas from growth-restricted pregnancies. Reduced IGF2 expression and loss of normal imprinting have been observed in a subset of FGR placentas. These findings suggest that dysregulation of imprinted genes may contribute to abnormal placental and fetal growth; however, the variability in reported methylation and imprinting patterns indicates that such abnormalities may not be consistently present across all FGR phenotypes [132,135].

### 6.2. Histone Modifications and Chromatin Remodeling in Placental Function

DNA methylation operates within a broader chromatin-regulatory system that includes post-translational histone modifications and ATP-dependent chromatin remodeling. Histone acetylation and methylation can modify chromatin accessibility and thereby influence the recruitment of transcriptional machinery. In the placenta, these mechanisms are relevant to the highly dynamic transitions between trophoblast proliferation, differentiation, invasion, and interaction with the maternal vasculature. Epigenetic regulation therefore provides a means by which developmental and environmental signals can be translated into changes in transcription without altering the underlying DNA sequence [19,130].

In FGR, changes in chromatin state have been investigated using genome-wide approaches, including chromatin accessibility and histone-mark profiling. Such studies support the presence of altered regulatory landscapes in growth-restricted placentas, with changes involving pathways related to hypoxia, angiogenesis, metabolism, and cellular differentiation [136]. Specifically, genome-wide mapping of active histone marks like H3K27ac has revealed widespread alterations in enhancer profiles near genes controlling microvascular expansion and tissue remodeling [137,138]. The biological significance of these findings is consistent with the concept that FGR is associated with coordinated changes in several placental regulatory systems rather than an isolated defect in a single signaling pathway [136]. Nevertheless, the direction and functional significance of individual histone changes may depend on placental cell type, gestational age, and the clinical subtype of FGR [135,137].

ATP-dependent chromatin remodeling complexes, including SWI/SNF-family complexes, provide another level of transcriptional regulation by altering nucleosome positioning and DNA accessibility [138]. Their potential importance in placental development is particularly relevant because trophoblast differentiation requires substantial and tightly regulated changes in gene expression [138,139]. Although direct evidence linking individual SWI/SNF components to human FGR remains more limited than the evidence for DNA methylation, integrated epigenomic studies increasingly indicate that altered chromatin accessibility can accompany changes in DNA methylation and transcription [126,140]. These observations support further investigation of chromatin remodeling as a mechanistic component of placental dysfunction [140,141].

### 6.3. Non-Coding RNAs and Post-Transcriptional Regulation in Healthy Pregnancy and FGR

Non-coding RNAs constitute an additional regulatory layer connecting genomic activity with placental phenotype. miRNAs can bind target mRNAs and regulate their stability or translation, whereas long non-coding RNAs (lncRNAs) may act through interactions with RNA molecules, transcriptional regulators, or chromatin-associated proteins. During normal placental development, these mechanisms participate in trophoblast proliferation, differentiation, migration, and responses to growth-factor signaling. The functional effects of miR-377, let-7a, and miR-145 on trophoblast growth and insulin-like growth factor-related signaling demonstrate how post-transcriptional regulation can modulate placental responses to developmental and maternal signals [19,142].

In FGR, differential miRNA expression has been associated with pathways involved in angiogenesis and nutrient transport. Altered placental miRNA expression has been identified in term FGR pregnancies, with predicted target genes implicated in angiogenic pathways and amino-acid transport. These findings are relevant to placental insufficiency, as impaired vascular adaptation and reduced nutrient transfer are key features of many FGR phenotypes [143].

Selective FGR has also been associated with coordinated changes in placental miRNA networks. Differentially expressed miRNAs have been identified in placentas from monochorionic twin pregnancies complicated by selective IUGR, with pathway analyses implicating TGF-β, MAPK, and Wnt signaling [144]. Evidence also supports the involvement of lncRNA–miRNA interactions in the molecular regulation of selective FGR [144]. Reduced MALAT1 expression has been observed in placental tissue from the smaller twin, with evidence of a regulatory relationship involving the miR-424/ERRγ/HSD17B1 axis, linking non-coding RNA regulation to trophoblast and endothelial functions [145]. These findings highlight the potential role of non-coding RNAs in integrating angiogenic, proliferative, and metabolic signals within the placenta [144,145].

The imprinted lncRNA H19 provides an additional connection between genomic imprinting and post-transcriptional regulation. H19 is located within the imprinted H19/IGF2 domain and has been implicated in placental growth regulation. Lower H19 expression has been observed in SGA placentas compared with AGA placentas in late normotensive pregnancy, accompanied by a tendency toward reduced IGF2 and decorin expression. These findings suggest that altered non-coding RNA expression may occur even in pregnancies complicated by FGR without major histopathological abnormalities, although the functional and causal significance of these associations requires further investigation [146].

### 6.4. Integrated Epigenomic Regulation and Implications for Placental Dysfunction in FGR

The available evidence supports an integrated model in which DNA methylation, genomic imprinting, histone modifications, chromatin accessibility, and non-coding RNAs interact to regulate placental function [19,130]. In normal pregnancy, these mechanisms permit coordinated adaptation of trophoblasts, placental vessels, and nutrient-transport systems to the changing requirements of the fetus [101,131]. In FGR, alterations in one regulatory layer may influence several others, potentially generating persistent changes in gene expression and cellular phenotype [134,143]. This framework is particularly relevant to pathways controlling angiogenesis, hypoxic adaptation, trophoblast differentiation, growth-factor signaling, inflammation, and nutrient transport [140,145].

Recent integrative methylome studies reinforce the concept that FGR-associated epigenetic signatures are distributed across multiple biological pathways rather than being restricted to a single locus [135]. Reproducible differences in DNA methylation have been identified in genes involved in vascular development, morphogenesis, immune regulation, and transcriptional control. Furthermore, multi-omics analyses of selective FGR, integrating DNA methylation, chromatin accessibility, and transcriptomic data, have revealed coordinated regulatory changes [134]. Such approaches may help distinguish primary molecular abnormalities from secondary consequences of placental hypoxia or altered cellular composition, although establishing causal relationships remains a major challenge [130,140].

From a translational perspective, placental epigenetic signatures may have potential value as biomarkers of placental dysfunction, as DNA methylation and non-coding RNA profiles can reflect molecular changes that precede or accompany the clinical manifestations of FGR [52,130]. However, the use of epigenetic markers for prediction or diagnosis requires careful consideration of tissue specificity, gestational age, placental cell composition, maternal exposures, fetal sex, and the distinction between constitutional smallness and pathological fetal growth restriction [131,135]. At present, the available evidence supports further investigation rather than the routine clinical application of individual placental epigenetic markers [19,134].

Overall, epigenetic and genomic regulation should be regarded as a central component of the molecular landscape governing placental function. The transition from normal pregnancy to FGR can be conceptualized not as the simple gain or loss of a single epigenetic mark, but rather as a disruption of interconnected regulatory networks. Future studies integrating methylome profiling, chromatin accessibility analysis, transcriptomics, proteomics, and single-cell approaches may help clarify which alterations are mechanistically involved in placental dysfunction and which represent adaptive or secondary responses. Such integrated analyses may ultimately facilitate the molecular stratification of FGR and the identification of placenta-specific therapeutic targets.

## 7. Metabolic Signaling and Nutrient Transport in Placental Function: From Homeostasis to FGR

Placental metabolism represents a dynamic interface between maternal nutrient availability and fetal growth. Rather than functioning as a passive conduit, the placenta senses oxygen, nutrients, hormones, and inflammatory signals and adjusts cellular metabolism, growth, and transplacental nutrient transfer accordingly. This adaptive capacity is particularly important because fetal growth depends not only on the amount of substrate reaching the maternal–fetal interface but also on the ability of trophoblast cells to sense, metabolize, and selectively transport these substrates. Insulin/IGF signaling, PI3K/AKT, mechanistic target of rapamycin (mTOR), AMP-activated protein kinase (AMPK), and related nutrient-sensing pathways therefore constitute an interconnected regulatory network linking placental growth to nutrient transport [20,131].

In a healthy pregnancy, this metabolic network permits the placenta to adapt progressively to the increasing demands of the fetus. mTOR signaling integrates amino-acid and energy availability with trophoblast protein synthesis and transporter activity, while insulin and IGF signaling provide additional endocrine inputs. Nutrient transporters, in turn, influence intracellular amino-acid and glucose availability and thereby feed back into metabolic signaling. The resulting system is better understood as a regulated circuit than as a collection of independent transport proteins. This network perspective is important for FGR because abnormalities in one component may propagate to other functional domains, including trophoblast growth, vascular adaptation, mitochondrial metabolism, and inflammatory signaling [21,115].

Evidence from human and experimental studies indicates that placental nutrient transport is altered in FGR. Reduced activity of amino acid transport systems, changes in transporter abundance or localization, and disturbances in nutrient-sensing pathways have all been reported. Importantly, these abnormalities do not necessarily occur to the same extent or in the same direction in all pregnancies complicated by FGR. Differences in gestational age, disease severity, fetal phenotype, maternal comorbidities, placental cell composition, and the distinction between early- and late-onset FGR may contribute to this heterogeneity. Accordingly, the most informative interpretation is not that FGR is characterized by a single “defective transporter”, but rather that placental nutrient acquisition and utilization may become uncoupled from fetal growth requirements [20,56,115].

### 7.1. mTOR and Nutrient-Sensing Pathways in Placental Homeostasis and FGR

The mTOR pathway is a central integrator of cellular nutrient and growth signals. In the placenta, mTORC1 and mTORC2 connect amino-acid availability, insulin/IGF signaling, cellular energy status, and protein synthesis with the regulation of nutrient transport. Placental mTOR signaling has been shown to be associated with amino-acid transporter activity and fetal growth, providing a mechanistic link between nutrient sensing and transplacental nutrient supply. The significance of this pathway extends beyond nutrient transport, as mTOR also influences trophoblast growth, differentiation, and metabolic adaptation [21,147].

In FGR, placental mTOR signaling is generally reduced, although the molecular phenotype may vary between clinical subtypes. Altered placental mTOR signaling has been reported in human FGR, while impaired mTOR activity has also been associated with reduced amino-acid transport. In this context, decreased mTOR activity may represent both a marker of placental nutrient stress and a potential mechanism contributing to insufficient nutrient transport capacity. This reciprocal relationship provides an important conceptual link between metabolic stress and placental insufficiency, rather than considering them as independent abnormalities [148].

The mTOR network also interacts with oxygen-sensing and cellular stress pathways. Nutrient deprivation, hypoxia, and oxidative or endoplasmic reticulum stress can converge on nutrient-sensing pathways, potentially reducing anabolic activity at a time when the placenta must maintain or increase its nutrient transport capacity. This interplay is consistent with the broader molecular model of FGR, in which vascular insufficiency, oxidative stress, inflammation, and metabolic dysfunction reinforce one another rather than acting as isolated processes [56].

### 7.2. System A Amino-Acid Transport and SNAT2

System A is a sodium-dependent amino-acid transport system that transfers small neutral amino acids, including glutamine, alanine, and serine, into trophoblast cells. SNAT2 (SLC38A2) is one of its principal transporters. System A is important not only because it contributes directly to the supply of amino acids to the fetal compartment, but also because intracellular amino-acid availability regulates trophoblast protein synthesis, cellular volume, and nutrient-sensing pathways. Thus, transporter activity links extracellular nutrient availability to intracellular metabolic state [20,149].

Reduced System A activity has been demonstrated in placental microvillous membrane preparations from pregnancies complicated by IUGR, providing direct functional evidence of impaired placental amino-acid transport [150]. Importantly, the magnitude of this reduction appears to be associated with the severity of fetal compromise, with particularly marked decreases reported in pregnancies with reduced umbilical blood flow [150,151,152]. Altered expression of the System A transporter SNAT2 has also been reported in IUGR placentas, with lower SNAT2 mRNA levels observed in pregnancies associated with reduced umbilical blood flow [153]. Together, these findings suggest that impaired System A-mediated amino-acid transport may be related not only to restricted fetal growth but also to the severity of underlying placental vascular dysfunction.

A critical point for interpretation is that transporter abundance does not necessarily reflect transporter function. SNAT2 activity depends not only on its overall expression but also on its trafficking to and retention within the trophoblast plasma membrane, processes regulated by mTOR signaling, ubiquitination, and cytoskeletal mechanisms. Consequently, measurements of transporter mRNA or total protein abundance alone may not fully capture functional impairment in placental amino-acid transport. This distinction supports a network-based interpretation of FGR, in which impaired nutrient transfer may arise from altered signaling, post-translational regulation, and transporter trafficking even when changes in total transcript or protein abundance are relatively modest [154,155,156].

The interaction between SNAT2 and mTOR signaling is particularly relevant to placental nutrient sensing. Amino acids transported through System A can contribute to intracellular amino-acid sensing and mTORC1 activation, while mTOR signaling regulates the activity and plasma-membrane trafficking of key amino-acid transporters, including SNAT2. In FGR, impaired mTOR signaling has been associated with reduced SNAT2 trafficking and System A activity, potentially limiting amino-acid availability to both the fetus and trophoblast cells. This bidirectional relationship provides a mechanistic framework in which disturbances in nutrient transport and intracellular nutrient signaling may reinforce one another, contributing to persistent metabolic dysfunction in placental insufficiency [148,154,157,158,159].

### 7.3. System L, LAT1, and Essential Amino-Acid Availability

System L provides a complementary transport route for large neutral amino acids, including essential amino acids such as leucine. LAT1 (SLC7A5) is a major System L transporter in trophoblasts. Unlike System A, System L operates largely as an exchanger and depends on the intracellular amino-acid pool. This functional relationship means that disturbances in one transport system can influence the performance of another, reinforcing the concept that placental nutrient transport is an integrated network [149].

Leucine is particularly important because it is both a substrate and a metabolic signal capable of influencing anabolic pathways, including mTORC1. Consequently, LAT1 occupies a strategic position at the interface of amino-acid transfer and trophoblast growth. Relationships between placental mTOR signaling and System L amino-acid transport have been demonstrated, supporting the interpretation that nutrient transport and intracellular signaling are mechanistically interconnected [21,147].

In FGR, placental amino-acid transport capacity is substantially reduced, with abnormalities reported across multiple transport systems, including System L [152]. Impaired nutrient transport is considered an important component of FGR pathophysiology, as altered transporter activity may limit the delivery of essential substrates to the fetus [56]. Importantly, these findings suggest that placental nutrient insufficiency reflects a broader disruption of coordinated nutrient-handling mechanisms rather than an isolated defect in a single transporter such as LAT1 [160].

### 7.4. Glucose Transport and Placental Metabolic Adaptation

Glucose is an essential substrate for both placental and fetal metabolism. GLUT1 (SLC2A1) is the predominant glucose transporter in human placental trophoblasts, while GLUT3 and other GLUT isoforms may contribute according to cellular context and metabolic demand. Placental glucose transfer is influenced by maternal glucose concentration, placental metabolism, oxygen availability, and fetal requirements, making glucose transport part of a broader adaptive system rather than a fixed process [20].

The relationship between placental glucose transport and FGR is more complex than a simple reduction in GLUT expression. Placental glucose uptake and utilization are influenced by maternal substrate availability, insulin signaling, oxygenation, and the metabolic demands of the placenta and fetus [161,162]. In addition, fetal glucose concentrations reflect not only placental glucose transfer but also fetal metabolic adaptation and glucose utilization [163]. Consequently, differences in GLUT expression should not automatically be interpreted as direct evidence of reduced transplacental glucose flux, particularly because the functional contribution of individual GLUT isoforms depends on their cellular localization and membrane distribution [162,164]. This distinction is important when integrating metabolic findings across different FGR phenotypes.

In some growth-restricted pregnancies, impaired placental perfusion and hypoxic or oxidative stress may alter glucose uptake and utilization without producing uniform changes in GLUT abundance [161,162]. Conversely, compensatory alterations in GLUT expression may occur in response to changes in maternal glucose availability or oxygenation. For example, increased placental GLUT3 expression has been reported in late-onset IUGR, suggesting that transporter regulation may represent an adaptive response to altered placental conditions rather than simply a marker of impaired transport [165]. The interpretation of GLUT biology therefore benefits from integrating transporter expression with functional glucose-transport measurements, placental glucose metabolism, maternal–fetal glucose concentration gradients, and clinical indicators of placental insufficiency [162].

### 7.5. Integration of Nutrient Transport with Oxygen, Inflammation, and Oxidative Stress

Nutrient transport cannot be separated from placental oxygenation and vascular function. The developing placenta normally experiences changing oxygen conditions, and physiological redox signaling participates in trophoblast differentiation and angiogenesis. When placental perfusion becomes inadequate, repeated hypoxia–reoxygenation can increase oxidative and nitrative stress, impair mitochondrial function, and alter trophoblast survival. Placental oxidative and nitrative stress in FGR may impair nutrient transfer while promoting trophoblast dysfunction, cellular senescence, and cell death. Thus, impaired nutrient transport may represent not only a consequence of placental vascular dysfunction but also a mechanism through which oxidative and metabolic stress further compromise placental function [166].

Inflammatory signaling provides another connection between metabolic and structural dysfunction. Cytokines and inflammatory mediators can modify trophoblast metabolism and transporter activity, while metabolic stress can itself activate inflammatory pathways. This reciprocal interaction is consistent with the broader concept of FGR as a network disorder involving angiogenic imbalance, inflammation, oxidative stress, and nutrient transport rather than a single pathogenic cascade [56].

The resulting model can be summarized as a functional feedback loop: impaired placental perfusion reduces oxygen and nutrient availability; cellular stress and redox imbalance alter trophoblast metabolism and nutrient sensing; mTOR and related signaling pathways become dysregulated; transporter trafficking and activity are impaired; and reduced nutrient transfer further limits the resources available to both the placenta and the fetus. This model provides a mechanistic link between the vascular abnormalities discussed in the preceding sections and the epigenetic and genomic regulatory mechanisms.

### 7.6. From Metabolic Signatures to Biomarker Development

The integrated metabolic model also has important implications for biomarker development. A single nutrient transporter is unlikely to capture the full biological heterogeneity of FGR, because placental nutrient transport is regulated not only by transporter abundance but also by upstream signaling pathways, oxygen and nutrient availability, cellular stress, and placental function [6,167,168]. In FGR, alterations in mTOR and other nutrient-sensing pathways, together with impaired amino-acid and glucose transport, occur in the context of abnormal placental perfusion and trophoblast dysfunction, supporting a multidimensional rather than single-marker approach [6,168]. A more informative strategy would therefore combine metabolic indicators with angiogenic markers such as placental growth factor (PlGF) and soluble fms-like tyrosine kinase-1 (sFlt-1), inflammatory markers, epigenetic signatures, and trophoblast- or placenta-derived extracellular vesicles, and interpret these molecular measurements alongside ultrasound and Doppler findings [12,169]. Evidence that sFlt-1/PlGF measurements may provide additional information when combined with ultrasound parameters further supports this integrative concept, although the clinical utility of multimarker strategies in FGR remains under investigation and requires prospective validation [57].

The potential value of multimodal profiling is supported by the increasing use of multi-omics approaches to characterize FGR placentas. Recent work emphasizes that FGR encompasses several interacting molecular programs, including impaired trophoblast invasion, angiogenic imbalance, oxidative stress, nutrient-transport failure, and epigenetic remodeling. Such findings support the development of molecularly stratified phenotypes, but they do not yet establish that any individual metabolic signature is sufficiently validated for routine clinical prediction [56,170].

From this perspective, the most innovative and clinically relevant contribution of metabolic research may lie not in identifying additional isolated transporter abnormalities, but rather in integrating transporter activity with the upstream signaling status of the placenta. Longitudinal studies combining maternal circulating biomarkers, placental molecular profiling, functional measurements of nutrient transport, and Doppler assessment may help determine whether metabolic abnormalities precede the clinical manifestation of FGR or primarily represent downstream consequences of established placental insufficiency.

## 8. Critical Appraisal and Future Research Directions

The evidence reviewed in this article supports a network-based interpretation of placental dysfunction in FGR, in which inflammatory signaling, extracellular matrix remodeling, angiogenic imbalance, epigenetic regulation, oxidative stress, and nutrient transport are functionally interconnected. Rather than identifying a single molecular abnormality as the universal cause of FGR, current findings suggest that different combinations of placental disturbances may converge on impaired maternal–fetal exchange. This framework is consistent with the multifactorial nature of placental dysfunction and the heterogeneity of FGR pregnancies [6,8]. Table 1 summarizes the molecular factors discussed, their physiological roles, and their contributions to FGR pathogenesis.

The strength of evidence, however, varies across pathways. Human placental studies provide clinically relevant associations, whereas cellular and animal models can offer mechanistic insights without necessarily establishing causality in human pregnancy. For example, NLRP3 inflammasome-related changes have been reported in human FGR placentas, while findings on MMP-9 suggest that its contribution to placental remodeling is context-dependent rather than uniformly detrimental [9,10,11]. Such observations require interpretation in light of the specific tissue, cellular compartment, and functional endpoint examined. Proposed compensatory vascular responses should therefore be treated as hypotheses unless directly demonstrated.

A central limitation is that many human studies analyze placental tissue collected at delivery, after FGR has already been diagnosed. Molecular differences observed at this stage cannot establish whether an alteration initiated placental dysfunction, arose secondarily from impaired perfusion or nutrient delivery, or represents an adaptive response to an adverse intrauterine environment. Reverse causation, tissue heterogeneity, confounding, and differences in analytical methods are particularly important when interpreting gene-expression and epigenetic profiles [18]. More broadly, the available evidence does not establish a single, sequential causal cascade applicable to all FGR pregnancies [45,51,73,128]. Changes in inflammatory signaling, angiogenesis, cell survival, and nutrient transport may be pathogenic, compensatory, or a combination of both, depending on disease severity, duration, gestational age, and the placental compartment examined [45,73,74,106,128,172]. Consequently, the direction of a molecular change alone does not indicate whether it drives dysfunction or reflects an attempt to preserve placental homeostasis.

One proposed temporal model is that abnormalities in early placentation or uteroplacental vascular remodeling precede impaired perfusion and cellular stress, which may then affect metabolic activity, nutrient transport, inflammatory signaling, and gene regulation [5]. Nevertheless, these processes may overlap, interact through feedback mechanisms, and vary by FGR onset, severity, and underlying placental pathology. Longitudinal studies and functional validation in appropriate experimental systems are needed to distinguish initiating mechanisms from downstream consequences and adaptive responses [51,128].

Epigenetic findings warrant similar caution. Differences in DNA methylation, histone modifications, genomic imprinting, chromatin accessibility, and non-coding RNA expression may contribute to altered placental function, but they may also arise in response to environmental, metabolic, or hypoxic stress [18,130,131,132,133,134,135,136]. Their interpretation is complicated by tissue composition, gestational age, environmental exposures, reverse causation, and study design. Accordingly, epigenetic signatures should be regarded as promising mechanistic and biomarker candidates rather than established causal determinants of FGR. Specific molecular claims should also match the endpoint directly measured: for example, WNT2 promoter methylation alone does not demonstrate transcriptional silencing without corresponding gene-expression evidence.

FGR is also biologically heterogeneous. Early-onset disease is more frequently associated with pronounced placental vascular dysfunction, whereas late-onset FGR may involve a greater contribution from metabolic disturbances and altered nutrient transport [51,128,165]. These are general tendencies rather than mutually exclusive mechanisms: vascular dysfunction, inflammation, altered metabolic signaling, and impaired nutrient transport can coexist in either phenotype. Gestational age at diagnosis alone is therefore insufficient to identify the predominant molecular mechanism in an individual pregnancy. Differences in disease severity, maternal conditions, placental composition, sampling procedures, and analytical methods may also contribute to inconsistent findings. In addition, changes in gene or protein abundance do not necessarily correspond to altered biological activity. This is particularly relevant to nutrient transport, where transporter function depends on membrane localization, trafficking, and upstream regulatory pathways, including mechanistic target of rapamycin (mTOR) signaling [148,154,155,156,157,158,159,162,164]. Molecular measurements should therefore be paired, where possible, with physiological or functional evidence.

The clinical utility of molecular biomarkers likewise depends on the marker, clinical setting, and intended application. The sFlt-1/PlGF ratio has established clinical utility primarily in the assessment of suspected pre-eclampsia; its role in FGR characterization and risk stratification must be interpreted in the context of clinical presentation and the available evidence [13,14]. Although it may provide complementary information in selected pregnancies complicated by placental dysfunction, it should not be considered a universally validated tool for classifying FGR phenotypes [13,14]. Circulating microRNAs, epigenetic signatures, and extracellular vesicle-associated markers remain promising research candidates, but their reproducibility, diagnostic and predictive performance, and incremental value over established clinical assessment require confirmation in adequately powered, independent cohorts [13,19,52]. Multimarker approaches may eventually refine FGR classification, but their clinical benefit has not yet been demonstrated.

The exposome provides a complementary framework for investigating the multifactorial origins of placental dysfunction. It encompasses environmental exposures across the life course, including those before and during pregnancy, and considers their interactions with maternal biology and genetic susceptibility [173]. Relevant exposures include nutrition and metabolic status, psychosocial stress, air pollution, environmental chemicals, and lifestyle-related factors. These may influence placental function through oxidative stress, inflammation, endocrine signaling, and epigenetic regulation [174]. The framework does not imply that any exposure inevitably causes FGR; rather, it highlights how cumulative and interacting influences may contribute to molecular heterogeneity. Integrating standardized exposure measurements with placental transcriptomic, epigenomic, and metabolomic profiling could help identify exposure-related signatures, although longitudinal studies are needed to distinguish causal effects from associations and account for exposure timing, intensity, and individual susceptibility.

Conventional bulk-tissue analyses may obscure differences between placental cell populations and anatomical regions. Single-cell and spatial transcriptomic approaches can help identify whether molecular changes are concentrated in particular trophoblast populations, vascular compartments, or regions of placental injury [8,124]. Their value will depend on rigorous sample characterization, independent replication, and validation against functional and clinical measurements. Spatially resolved associations alone cannot establish causality or demonstrate that a candidate therapeutic target can be safely manipulated during pregnancy.

**Figure 8:**
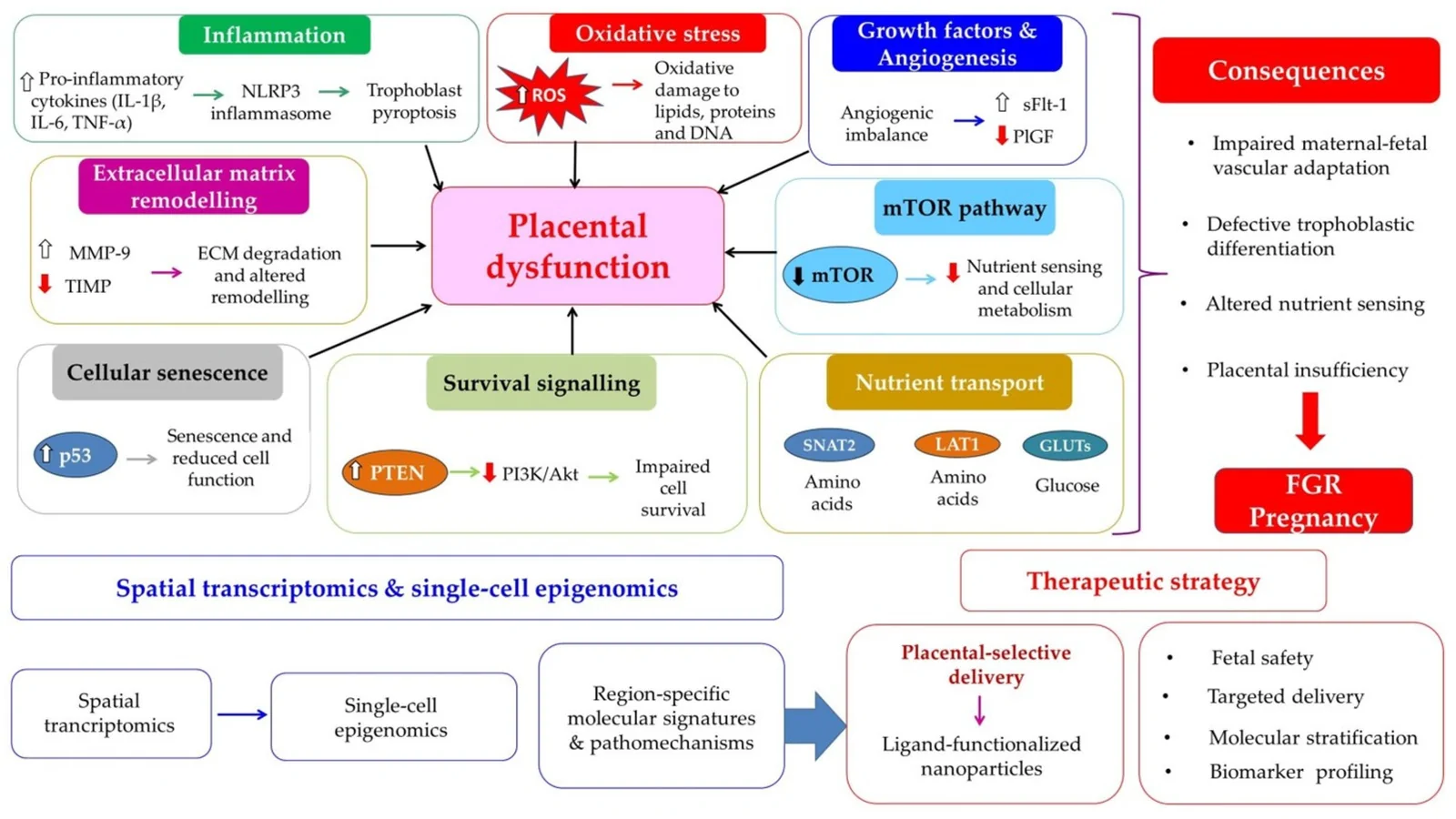
Integrated molecular network linking placental dysfunction to FGR. The schematic illustrates the interconnected roles of inflammation, oxidative stress, extracellular matrix remodeling, angiogenic imbalance, cellular senescence, cell survival signaling, mTOR-dependent nutrient sensing, and placental nutrient transport in the pathophysiology of FGR. These mechanisms converge to impair trophoblast function, disrupt maternal–fetal vascular adaptation, alter nutrient availability, and ultimately contribute to placental insufficiency. Furthermore, spatial transcriptomics and single-cell epigenomics provide a framework for molecular stratification and the development of placenta-selective therapeutic strategies.

Future research should integrate molecular biomarkers with established clinical assessments, including fetal growth trajectories and Doppler findings. Combining angiogenic markers with inflammatory, metabolic, and epigenetic measurements may help define biologically distinct FGR phenotypes and determine whether molecular profiles provide clinically meaningful information beyond current approaches [12,13,14,167,168,169]. Priorities include prospective studies with clearly defined phenotypes, standardized sampling and analytical procedures, and independent validation cohorts. Where feasible, molecular measurements should be paired with functional assays to determine whether observed changes correspond to impaired transport, altered signaling, or other measurable aspects of placental dysfunction. Serial fetal growth assessments, Doppler measurements, and appropriately validated maternal circulating biomarkers may help characterize disease progression, while human placental explants, trophoblast cultures, organoid systems, and well-characterized animal models can test temporal relationships and the effects of targeted pathway manipulation. These experimental systems provide complementary evidence but should not be assumed to reproduce all aspects of human FGR. Figure 8 summarizes the integrated molecular framework, linking placental stress signals and downstream functional consequences with emerging biomarker and therapeutic strategies.

Therapeutic translation remains a longer-term objective. Inflammatory, angiogenic, metabolic, and epigenetic pathways may offer targets for investigation, but their functions extend beyond the placenta and can involve essential maternal and fetal processes. Candidate interventions therefore require rigorous assessment of target specificity, efficacy, and maternal–fetal safety. Nanocarrier-based delivery is one investigational approach. Megalin-targeting liposomes have been explored for placenta-directed drug delivery [175], while folate-functionalized polymeric nanoparticles, including PLGA–PEG formulations, have shown enhanced placental tissue accumulation in experimental transport studies using placental cell models and ex vivo human placental perfusion [176]. These findings support further study but do not establish therapeutic efficacy in FGR. Biodistribution, targeting specificity, maternal and fetal safety, and potential long-term effects must be evaluated before clinical translation.

Overall, the principal research priority is to move beyond descriptive molecular associations toward reproducible, functionally validated, and clinically informative evidence. Harmonized FGR phenotyping, prospective longitudinal cohorts, the integration of molecular and physiological measurements, and independent validation will be essential to determine which molecular signatures can improve risk stratification and which pathways warrant further therapeutic investigation. Until such evidence becomes available, molecular profiling should be regarded as a tool for refining our biological understanding and generating testable hypotheses, rather than as a replacement for established clinical surveillance or individualized decisions regarding the timing of delivery.

### 8.1. Translational Implications for Biomarker Development and Mechanism-Based Therapeutic Interventions

The integrated molecular framework presented in this review shifts the pathophysiological paradigm from single-pathway abnormalities to an integrated, multidimensional network operating at the maternal–placental–fetal interface. The structural and functional failures characterising placental insufficiency do not arise from isolated genetic or environmental insults; rather, they represent a coordinated cascade where immunologic, angiogenic, growth-regulatory, epigenetic, and metabolic pathways continuously reinforce one another. Understanding how these systems converge is essential for transitioning from purely observational clinical surveillance to targeted, mechanism-based therapeutic strategies [5,56].

One of the most immediate translational opportunities lies in the expansion of circulating biomarker panels. The angiogenic imbalance represented by increased sFlt-1 and reduced PlGF, and particularly the maternal sFlt-1/PlGF ratio, has already demonstrated substantial clinical potential for identifying placental insufficiency and differentiating pathological FGR from constitutional smallness [12,14,116,177]. However, angiogenic biomarkers primarily capture one component of placental dysfunction and may not adequately reflect the inflammatory, epigenetic, metabolic, and trophoblast-specific abnormalities described in this review. Consequently, future biomarker development may benefit from combining circulating angiogenic factors with inflammatory mediators, placental-derived microRNAs, cell-free nucleic acids, and molecular signatures related to trophoblast function and nutrient transport.

Placental DNA methylation profiles have already been associated with fetal growth phenotypes, providing proof-of-principle that epigenetic signatures may capture clinically relevant alterations in placental biology [18,133]. Similarly, systematic evidence indicates that specific placental and circulating miRNA profiles are associated with FGR and regulate pathways involved in angiogenesis and PI3K/AKT signaling, although their clinical utility requires validation in longitudinal maternal blood samples and well-characterized FGR populations [143,178]. More recently, integrative analyses combining chromatin accessibility, DNA methylation, and transcriptomic data have demonstrated coordinated epigenetic and transcriptional alterations in FGR placentas, supporting the concept that multi-omics signatures may provide a more comprehensive representation of disease biology than individual biomarkers [1]. Thus, the future biomarker framework should move toward longitudinal, multimodal panels integrating circulating molecular signals with ultrasound and Doppler parameters, allowing molecular stratification of FGR phenotypes and potentially improving the distinction between predominantly vascular, inflammatory, and metabolic forms of placental dysfunction.

The same network-based concept has direct implications for therapeutic development. Rather than targeting a single downstream consequence of placental insufficiency, mechanism-based interventions could be designed to restore specific dysfunctional molecular nodes according to the biological phenotype of each pregnancy. Experimental evidence supporting increased placental NLRP3 signaling in FGR provides a rationale for investigating selective inflammasome inhibition as a means of reducing inflammation-associated trophoblast injury, although such approaches remain preclinical [9]. Similarly, restoration of placental angiogenic signaling through targeted delivery of growth factors or other molecular regulators has been investigated experimentally, with placental gene therapy representing a potential strategy for achieving localized rather than systemic modulation of placental pathways [179].

The metabolic network offers several potential therapeutic entry points, including restoration of nutrient-sensing pathways, modulation of placental transporter activity, and reduction in oxidative or inflammatory stress. Preclinical studies suggest that restoring blunted trophoblast mTOR signaling can restore downstream transporter trafficking and the surface localization of vital amino acid transport proteins such as SNAT2 and LAT1 [21,147]. However, the biological importance of mTOR, amino-acid transport, glucose metabolism, and redox signaling during normal fetal development creates substantial safety constraints [20]. Manipulating these pathways systemically could affect both maternal physiology and fetal development, making placenta-selective strategies particularly important. Current evidence supports continued investigation of mechanism-based interventions rather than routine clinical use of metabolic pathway modulators for FGR. Effective therapies capable of selectively restoring placental nutrient transport remain limited, and experimental strategies require validation in appropriately phenotyped human pregnancies [56,92]. The translational priority is therefore to determine which molecular abnormalities are causal, which are compensatory, and at what gestational stage intervention could restore placental function without disrupting normal developmental signaling.

### 8.2. Future Therapeutic Perspectives: Preclinical Evidence and Translational Challenges

Despite advances in understanding the molecular mechanisms underlying placental dysfunction in FGR, no established prenatal pharmacological treatment currently reverses the underlying condition. Delivery remains the principal clinical intervention when continued pregnancy poses an unacceptable risk to the fetus. Consequently, therapeutic strategies targeting placental dysfunction should be considered investigational until their efficacy and safety have been demonstrated in appropriately designed studies.

Preclinical investigations have evaluated several approaches, including antioxidant supplementation, vasodilators, nutritional interventions, and immunomodulatory strategies. However, a systematic review of prenatal interventions in animal models identified substantial methodological limitations, including frequent risks of bias and limited reporting of adverse effects and long-term offspring outcomes. Although many studies reported improvements in fetal survival or birthweight, these findings should be interpreted cautiously and cannot be directly extrapolated to clinical benefit in human pregnancies [180].

Placenta-targeted gene delivery represents a further experimental approach. Preclinical studies have investigated the delivery of growth-related factors, including IGF-1, to modify placental function and nutrient transport. Reviews of this work describe promising findings in selected animal and human placental experimental models, while also highlighting unresolved questions concerning delivery efficiency, duration of transgene expression, off-target effects, immunogenicity, and potential maternal or fetal toxicity. These approaches therefore remain at the preclinical stage and require additional validation before clinical application can be considered [1,180,181].

Similarly, interventions directed at inflammatory signaling, angiogenic pathways, epigenetic regulators, or non-coding RNAs may provide opportunities for future investigation. Nevertheless, the identification of a pathway associated with FGR does not establish that its therapeutic modulation will improve pregnancy outcomes. Because many of these pathways participate in normal placental development and fetal organogenesis, interventions must demonstrate target specificity and a favorable safety profile in relevant experimental models.

Future preclinical studies should therefore prioritize reproducibility, appropriate control groups, randomization and blinding, dose optimization, and systematic assessment of maternal, placental, fetal, and postnatal outcomes. Where feasible, findings should be validated across complementary animal and human placental models. In particular, studies of targeted delivery systems should establish both therapeutic activity at the placental target and the extent of unintended fetal exposure.

Overall, these approaches represent promising research directions rather than established treatments for FGR. Progress toward clinical translation will depend on rigorous demonstration of their efficacy, reproducibility, and maternal–fetal safety, followed by carefully designed clinical trials. Until such evidence becomes available, these proposed molecular therapies should not be considered clinically validated alternatives to established fetal surveillance strategies and individualized obstetric management.

### 8.3. Unified Spatial Transcriptomics and Single-Cell Epigenomics: Mapping the Microenvironmental Topology of FGR

A major limitation of historical placental research in FGR has been its reliance on bulk tissue profiling. By homogenizing whole placental explants, distinct cell-type variations and localized microenvironmental gradients were systematically masked. The true originality of the current molecular landscape lies in the conceptual integration of spatial transcriptomics and single-cell epigenomics (scATAC-seq/scRNA-seq), which shifts the paradigm from global placental failure to a high-resolution, topologically mapped network disorder [8,124].

Emerging spatial profiling demonstrates that placental insufficiency is profoundly regionalized. Under FGR conditions, specific anatomical zones—particularly the proximal anchoring villi and the perimeter of defective maternal spiral arteries—exhibit distinct “hotspots” of chromatin closure and translational arrest [90]. Single-cell multi-omics reveal that extravillous trophoblasts (EVTs) invading these ischemic niches undergo focal epigenetic metaplasia, characterized by localized hypermethylation of the WNT2 promoter and downstream suppression of matrix-remodeling cascades managed by MMP-9 [17]. Conversely, neighboring normoxic compartments often demonstrate hyper-localized compensatory responses, such as the site-specific upregulation of pro-angiogenic splice variants like IGF-IEa and IGF-IEb within the endothelium lining of intact fetal vessels [102,103].

Furthermore, this spatial deconvolution helps map the cellular distribution of the pro-inflammatory shift. Hyperactivation of the NLRP3 inflammasome and subsequent gasdermin-D-mediated pyroptosis are not uniformly distributed throughout the syncytiotrophoblast layer but appear to be anatomically associated with areas of severe local shear stress and persistent maternal vascular malperfusion [9,50]. Mapping these localized cellular interactions provides a novel framework for therapeutic development. Rather than relying on systemic administration of broad metabolic or angiogenic modulators, which may carry substantial risks of transplacental fetal toxicity, future interventions could employ target-selective, ligand-functionalized nanoparticles designed to navigate the placental vasculature and release their molecular payload specifically within these epigenetically compromised microenvironments [114,182,183].

## 9. Conclusions

The synthesis of molecular and clinical evidence presented in this review underscores a fundamental paradigm shift: placental dysfunction in FGR does not result from an isolated genetic, immune, or vascular defect but rather reflects a highly integrated, self-reinforcing pathological network. As outlined throughout this review, chronic inflammatory activation and NLRP3-mediated trophoblast pyroptosis directly compromise the tissue architecture required for matrix metalloproteinase-driven spiral artery remodeling. This initial architectural disruption contributes to the profound angiogenic imbalance characterized by the sFlt-1/PlGF axis, which, in turn, induces downstream endothelial dysfunction and accelerates intrinsic trophoblast apoptosis. Ultimately, the resulting structural and vascular impairment disrupts the placenta’s key metabolic sensing mechanisms, particularly the PI3K/AKT/mTOR signaling pathway, leading to the marked downregulation and impaired trafficking of essential amino acid transporters (SNAT2 and LAT1) and glucose transporters (GLUTs).

Crucially, this structural and metabolic breakdown is orchestrated and sustained by profound epigenetic and genomic dysregulation. The dynamic placental methylome, together with specific histone modifications and restricted chromatin accessibility mediated by regulatory subunits such as ARID1A, acts as a functional interface through which environmental, maternal, and hypoxic stressors are translated into long-term transcriptional silencing. In conjunction with complex post-transcriptional regulatory networks involving microRNAs and long non-coding RNAs, these epigenetic alterations may lock the placenta into a state of chronic functional insufficiency, compromising the essential nutrient supply required for the fetus to achieve its full growth potential.

Looking ahead, this interconnected landscape underscores the inherent limitations of traditional clinical interventions targeting single molecular pathways. The transition from passive clinical surveillance to active, mechanism-based management of FGR will require biomarker-guided molecular stratification, distinguishing early-onset vascular dysfunction from late-onset metabolic and nutrient transport abnormalities. The development of targeted, cell-specific therapeutics, such as localized growth factor gene delivery, microRNA modulators, selective inflammasome inhibitors, and stem cell-derived extracellular vesicles, holds considerable promise for restoring placental homeostasis. However, translating these approaches into clinical practice requires rigorous ethical and pharmacological evaluation to minimize the risk of transplacental fetal toxicity and developmental disturbances. Until multi-omics profiling is systematically integrated with functional human organoid models through large-scale collaborative research networks, precise clinical staging and optimization of delivery timing will remain the most effective strategies for managing this serious pregnancy complication.

## Figures and Tables

**Table 1:** Integrated overview of molecular pathways associated with FGR: Physiological Roles, Pathophysiological Alterations, and Evidence from Placental Tissue, Maternal Blood, and Cord Blood.

Molecular Factor/Pathway	Principal Physiological Role in the Placenta	Alterations Associated with FGR	Potential Contribution to Placental Dysfunction	Evidence in FGR (Placenta, Maternal Serum, Cord Blood)	Ref.
**Inflammatory cytokines (IL-1β, IL-6, TNF-α)**	Participate in controlled immune adaptation at the maternal–fetal interface.	Increased inflammatory signaling has been associated with placental dysfunction and FGR.	Chronic inflammation can impair trophoblast function, vascular remodeling, and endothelial integrity.	**Placenta:** Evidence of increased inflammatory signaling. **Maternal blood:** Cytokine alterations have been reported in FGR, although results vary between studies. **Cord blood:** Inflammatory biomarkers may be altered, but findings are heterogeneous.	[166]
**Adipokines (adiponectin, apelin, leptin, visfatin, resistin, PAI-1, chemerin, FABP-4)**	Regulate placental energy metabolism, nutrient transport, angiogenesis, endothelial function, and inflammatory homeostasis.	Reduced adiponectin and apelin, and increased leptin, visfatin, resistin, PAI-1, chemerin, and FABP-4 have been reported in association with FGR-related placental dysfunction.	A shift toward a pro-inflammatory and pro-oxidative adipokine profile may promote endothelial dysfunction, impaired placental signaling, and inadequate fetal growth.	**Placenta:** Altered adipokine expression/concentrations have been reported. **Maternal blood:** Reduced adiponectin and apelin and increased pro-inflammatory adipokines have been reported. **Cord blood:** Alterations have also been described, but evidence is less uniform than for maternal serum and placenta.	[35,36,37,38]
**NLRP3 inflammasome**	Innate immune multiprotein complex that activates caspase-1, promoting the maturation and secretion of IL-1β and contributing to placental inflammatory signaling.	Increased placental NLRP3 activation, caspase-1, IL-1β, and IL-18 have been reported in FGR.	Promotes inflammatory signaling and trophoblast pyroptosis, potentially affecting proliferation, invasion, and placental vascular remodeling.	**Placenta:** Increased NLRP3 inflammasome expression/activation has been demonstrated in FGR placental tissue, with increased NLRP3, caspase-1, and downstream inflammatory signaling. **Maternal blood:** Evidence for circulating NLRP3-related changes is limited; NLRP3 is not an established circulating FGR biomarker. **Cord blood:** Direct evidence of NLRP3 inflammasome activation is limited; NLRP3 itself is not sufficiently validated as a cord-blood biomarker.	[9]
**MMP-9/TIMP system**	Supports extracellular matrix degradation/remodeling, extravillous trophoblast invasion, spiral-artery remodeling, and modification of angiogenic factors.	Placental MMP-9 expression/activity may be reduced, whereas some studies report increased circulating MMP-9 in FGR.	Disturbed MMP-9/TIMP-1 activity may impair trophoblast invasion, extracellular matrix turnover, and spiral-artery remodeling.	**Placenta:** Human evidence indicates altered MMP-9 expression/activity in FGR. **Maternal blood:** Increased circulating MMP-9 has been reported in some FGR cohorts. **Cord blood:** Not established as a validated FGR biomarker.	[10,11,58]
**VEGF/PlGF**	Promote placental angiogenesis, endothelial proliferation, and development of the villous vascular network.	Reduced pro-angiogenic activity and altered circulating concentrations, particularly of PlGF, are associated with placental insufficiency.	Impaired angiogenesis and endothelial function may contribute to inadequate uteroplacental perfusion.	**Placenta:** Altered angiogenic signaling has been documented. **Maternal blood:** Strong evidence supports altered PlGF and VEGF-related angiogenic balance. **Cord blood:** Less consistently investigated as a clinical marker of FGR.	[12,14]
**TGF-β signaling**	Regulates trophoblast differentiation and invasion, extracellular matrix remodeling, immune adaptation, and aspects of vascular development.	Altered TGF-β2 expression has been reported in placental and placental-bed tissues in FGR.	May contribute to abnormal trophoblast invasion, impaired spiral-artery remodeling, and altered placental architecture.	**Placenta:** Evidence of altered TGF-β2 expression/localization in FGR. **Maternal blood:** Limited direct evidence for TGF-β2 as an FGR biomarker. **Cord blood:** Evidence exists for TGF-β1, but this should not be extrapolated directly to TGF-β2.	[82]
**IGF/IGFBP system**	Regulates trophoblast proliferation, differentiation, survival, angiogenesis, and nutrient transport; IGFBPs control IGF bioavailability.	Reduced IGF-1 and altered placental IGF-2/IGFBP expression have been reported in FGR; IGFBP-1/2 may increase, whereas IGFBP-3 may decrease.	Reduced IGF bioactivity may impair trophoblast growth, increase apoptosis, disturb angiogenesis, and reduce nutrient transport.	**Placenta:** Direct evidence of altered IGF/IGFBP signaling in FGR. IGF-IEa and IGF-IEb show isoform- and compartment-specific protein-level changes, with reduced IGF-IEa in extravillous trophoblasts but increased IGF-IEa/IEb immunoreactivity in selected placental vascular and trophoblast compartments, despite largely unchanged mRNA expression. **Maternal blood:** Increased IGFBP-1/2 and reduced IGF activity have been reported. **Cord blood:** Reduced IGF-1 is associated with lower birth weight and more severe FGR.	[92,98,99]
**sFlt-1**	Soluble decoy receptor that binds VEGF and PlGF and regulates their bioavailability.	Increased maternal circulating sFlt-1 and an elevated sFlt-1/PlGF ratio are associated with FGR, particularly placental FGR.	Reduces VEGF/PlGF bioavailability, contributing to endothelial dysfunction and impaired uteroplacental perfusion.	**Placenta:** Increased placental production is implicated in angiogenic imbalance. **Maternal blood:** Strong evidence supports increased sFlt-1/PlGF ratio in FGR compared with AGA. **Cord blood:** Not routinely used or sufficiently validated as an FGR biomarker.	[12,117]
**p53 pathway**	Regulates cell-cycle arrest, DNA repair, apoptosis, and cellular responses to hypoxia and stress.	Increased p53 expression is associated with increased trophoblast apoptosis in FGR placentas.	Excessive apoptosis may impair trophoblast survival and placental cellular homeostasis.	**Placenta:** Direct human evidence of increased p53 in FGR trophoblasts. **Maternal blood:** Insufficient evidence for routine assessment in FGR. **Cord blood:** Insufficient evidence for p53 as a validated FGR biomarker.	[119]
**PTEN/PI3K–AKT axis**	PTEN is a lipid phosphatase and negative regulator of PI3K/AKT signaling; this axis modulates trophoblast proliferation, migration, invasion, survival, differentiation, and placental vascular remodeling.	Dysregulation of the PTEN/PI3K/AKT axis may occur in FGR; reduced PI3K/AKT phosphorylation has been reported in human FGR placentas.	Altered PTEN-mediated inhibition of PI3K/AKT may impair trophoblast proliferation, migration, invasion, and survival and may contribute to abnormal vascular remodeling and placental insufficiency.	**Placenta:** Altered PTEN-related signaling is supported, but consistent PTEN overexpression is not established. **Maternal blood:** PTEN is not an established circulating FGR biomarker. **Cord blood:** No validated clinical biomarker evidence.	[123,125,171]
**ARID1A/SWI–SNF**	ARID1A is a core regulatory subunit of the SWI/SNF chromatin-remodeling complex, regulating chromatin accessibility and transcriptional programs involved in trophoblast differentiation, proliferation, survival, invasion, and cellular stress responses.	Altered ARID1A expression has been reported in FGR placentas, including increased nuclear expression in syncytiotrophoblasts and reduced expression in stem-villus veins in late FGR.	Altered ARID1A activity may disturb trophoblast differentiation, proliferation, and invasion and may influence placental responses to chronic insufficiency; its specific causal contribution to FGR remains uncertain.	**Placenta:** Human evidence indicates altered ARID1A expression in IUGR/FGR placental compartments. **Maternal blood:** No established ARID1A FGR biomarker. **Cord blood:** No established clinical evidence. Mechanistic evidence remains limited.	[139]
**Epigenetic marks (DNA methylation, histone marks)**	Provide a regulatory interface between environmental/maternal influences and gene-expression programs.	Aberrant global DNA methylation, discrete changes in promoter H3K27ac, and altered chromatin accessibility have been reported.	May alter expression of angiogenic, structural, and metabolic/nutrient-transport networks.	**Placenta:** Multi-omics studies support epigenomic reprogramming in selected FGR cases. **Maternal/cord blood:** Cell-free DNA signatures are under investigation.	[135,153]
**Non-coding RNAs (miRNAs, lncRNAs)**	Provide post-transcriptional regulation and coordinate target-transcript networks.	Altered expression or sorting of specific miRNAs (e.g., miR-424, miR-15b, miR-146a) and reduced expression of protective lncRNAs (e.g., H19, MALAT1) have been reported.	May jointly regulate networks involved in capillary growth, inflammatory control, and nutrient transfer.	**Placenta:** Profiling supports tissue-specific disruption of miRNA–mRNA networks. **Maternal/cord blood:** Circulating non-coding RNA signatures show potential as early predictive markers, but further validation is needed.	[52,143,146]
**Nutrient transporters (SNAT2, LAT1, GLUT isoforms)**	Mediate uptake and maternal–fetal transfer of essential amino acids and glucose.	Reduced activity and altered membrane trafficking of SNAT2 (System A) and LAT1 (System L), as well as altered expression of GLUT1, GLUT3, and GLUT4, have been reported.	Reduced transporter activity may limit delivery of substrates required for fetal growth and development.	**Placenta:** Reduced transporter function has been reported, particularly in pregnancies with severely compromised Doppler indices. **Maternal/cord blood:** These transporters are not established as circulating FGR biomarkers.	[149,152,153]

(Abbreviations: AGA, appropriate for gestational age; ARID1A, AT-rich interaction domain 1A; DNA, deoxyribonucleic acid; ECM, extracellular matrix; FABP-4, fatty acid-binding protein 4; FGR, fetal growth restriction; GLUT, glucose transporter; H3K27ac, histone H3 lysine 27 acetylation; IGF, insulin-like growth factor; IGFBP, insulin-like growth factor-binding protein; IL-1β, interleukin-1 beta; IL-6, interleukin-6; LAT1, L-type amino acid transporter 1; lncRNA, long non-coding RNA; MALAT1, metastasis-associated lung adenocarcinoma transcript 1; miRNA, microRNA; MMP-9, matrix metalloproteinase-9; mRNA, messenger RNA; mTOR, mechanistic target of rapamycin; NLRP3, NLR family pyrin domain containing 3; PAI-1, plasminogen activator inhibitor-1; PI3K, phosphoinositide 3-kinase; PlGF, placental growth factor; PTEN, phosphatase and tensin homolog; RNA, ribonucleic acid; SNAT2, sodium-coupled neutral amino acid transporter 2; sFlt-1, soluble fms-like tyrosine kinase-1; SWI/SNF, switch/sucrose non-fermentable chromatin-remodeling complex; TGF-β, transforming growth factor beta; TIMP-1, tissue inhibitor of metalloproteinases 1; TNF-α, tumor necrosis factor alpha; VEGF, vascular endothelial growth factor).

## Data Availability

This is a review, and all the data are published and cited in the text.
